# Integrating Single‐Cell and Spatial Transcriptomics Reveals Heterogeneity of Early Pig Skin Development and a Subpopulation with Hair Placode Formation

**DOI:** 10.1002/advs.202306703

**Published:** 2024-04-01

**Authors:** Yi Wang, Yao Jiang, Guiyan Ni, Shujuan Li, Brad Balderson, Quan Zou, Huatao Liu, Yifan Jiang, Jingchun Sun, Xiangdong Ding

**Affiliations:** ^1^ State Key Laboratory of Animal Biotech Breeding National Engineering Laboratory for Animal Breeding Laboratory of Animal Genetics Breeding and Reproduction Ministry of Agriculture and Rural Affairs College of Animal Science and Technology China Agricultural University Beijing 100193 China; ^2^ Division of Genetics and Genomics Institute for Molecular Bioscience The University of Queensland Brisbane 4072 Australia; ^3^ School of Chemistry & Molecular Biosciences The University of Queensland Brisbane 4067 Australia; ^4^ Key Laboratory of Animal Genetics Breeding and Reproduction of Shaanxi Province Laboratory of Animal Fat Deposition & Muscle Development College of Animal Science and Technology Northwest A&F University Yangling 712100 China

**Keywords:** early skin development, hair follicle, hair placode formation, pigs, single‐cell RNA sequencing, spatial transcriptome

## Abstract

The dermis and epidermis, crucial structural layers of the skin, encompass appendages, hair follicles (HFs), and intricate cellular heterogeneity. However, an integrated spatiotemporal transcriptomic atlas of embryonic skin has not yet been described and would be invaluable for studying skin‐related diseases in humans. Here, single‐cell and spatial transcriptomic analyses are performed on skin samples of normal and hairless fetal pigs across four developmental periods. The cross‐species comparison of skin cells illustrated that the pig epidermis is more representative of the human epidermis than mice epidermis. Moreover, Phenome‐wide association study analysis revealed that the conserved genes between pigs and humans are strongly associated with human skin‐related diseases. In the epidermis, two lineage differentiation trajectories describe hair follicle (HF) morphogenesis and epidermal development. By comparing normal and hairless fetal pigs, it is found that the hair placode (Pc), the most characteristic initial structure in HFs, arises from progenitor‐like *OGN* ^+^/*UCHL1* ^+^ cells. These progenitors appear earlier in development than the previously described early Pc cells and exhibit abnormal proliferation and migration during differentiation in hairless pigs. The study provides a valuable resource for in‐depth insights into HF development, which may serve as a key reference atlas for studying human skin disease etiology using porcine models.

## Graphical abstract

Pigs are a valuable resource for human medical research. This study presents a high resolution spatial‐temporal transcriptome atlas to study embryonic pig skin. The results indicate notable similarities between pigs and humans in early skin development. Furthermore, by comparing normal and hairless pig fetuses, the study reveals a cell subpopulation causing lower hair placode density and further hairless in pigs.

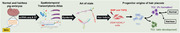

## 1 Introduction

The skin is the largest organ of the body and plays several vital roles, such as protection against environmental factors (biological, physical, or chemical), thermoregulation, metabolism, and sensation.^[^ ^1^ ^]^ Due to its scarcity and ethical concerns regarding its use, human skin, especially fetal skin, remains mostly unavailable for research on complex congenital skin genetic diseases. Therefore, mammals, rodents, and reptiles are considered suitable surrogates for human skin research. However, the skin of most animals, including rats, mice, guinea pigs, dogs, rabbits, and other non‐primates, show marked anatomic differences from human skin, for example, a thin epidermis,^[^ ^2^ ^]^ loose dermal structures,^[^ ^3^ ^]^ underdeveloped vascular system,^[^ ^4^ ^]^ and contraction heals in wound healing.^[^ ^5^ ^]^ Researchers have discovered that pig skin is an excellent alternative to human skin and is widely utilized as a substitute in various fields of dermatological research. This is primarily due to its anatomical and physiological similarities to human skin.^[^ ^6^ ^]^ In terms of skin appendages, the hair follicle (HF) exhibits remarkable similarities in cell types and morphogenic development between humans and pigs.^[^ ^7^ ^]^ Moreover, we have shown that the timing of HF initiation, characteristic markers (hair placode), and key genes involved in porcine embryonic HF morphogenesis are similar to those reported in humans.^[^ ^7^ ^]^ Considering their accessibility and suitability, pigs are potential animal models for human skin and hair research, particularly for complex congenital diseases.

The morphogenesis of HFs comprises three stages: induction, organogenesis, and cytodifferentiation. The initial observable structure in HF morphogenesis is the hair placode (Pc) at the induction stage.^[^ ^8^ ^]^ The formation of HFs in developing embryonic skin involves stepwise signaling between the epidermal placode and mesenchymal dermis through coordinated signaling pathways, including Wnt, Sonic Hedgehog (SHH), Ectodysplasin A (EDA), Notch, and Bone Morphogenetic Protein (BMP) pathways.^[^ ^9^ ^]^ While numerous genes, including *EDAR*,^[^ ^10^ ^]^ *SOX9*,^[^ ^11^ ^]^ *LEF1*,^[^ ^12^ ^]^ *Wnt10b*,^[^ ^13^ ^]^ *DKK4*,^[^ ^14^ ^]^ and *BMP4* ^[^ ^15^ ^]^ have been established as regulators of Pc formation, the precise spatiotemporal dynamics of their regulatory role during development remain unknown. Understanding the molecular events underlying the spatial and temporal aspects of Pc formation is crucial for replicating these processes in vitro, enabling more comprehensive mechanistic studies of hair growth.^[^ ^16^ ^]^

Single‐cell RNA sequencing (scRNA‐seq) technologies have enabled the profiling of a large number of cells to comprehensively dissect the cellular composition of complex tissues.^[^ ^17^ ^]^ ScRNA‐seq is, therefore, a powerful tool for understanding the cellular and molecular mechanisms of development at a high resolution and has been utilized to study early skin development^[^ ^18^ ^]^ and HF morphogenesis^[^ ^19^ ^]^ in mice. However, scRNA‐seq does not reflect the spatial organization of cells because of tissue disassociation during sample preparation. Spatial transcriptomics (ST) overcomes this limitation by sequencing RNA from intact tissues and labeling each RNA molecule according to the spatial location from which it was sampled.^[^ ^20^ ^]^ The Visium ST platform also captures the cell morphology by hematoxylin and eosin (H&E) staining of the same tissue sample. ST has been utilized in studies on the heart,^[^ ^21^ ^]^ brain,^[^ ^22^ ^]^ intestine,^[^ ^23^ ^]^ kidneys,^[^ ^24^ ^]^ cancers,^[^ ^25^ ^]^ and adult skin.^[^ ^26^ ^]^ However, to date, ST has not been used to construct molecular atlases of embryonic skin or to investigate HF development.

Therefore, in this study, we sought to establish a dynamic cell atlas of skin development during pig fetal development to identify the first signaling cascade initiating HF morphogenesis, Pc formation, and the gene expression profiles of Pc progenitor cells. We performed scRNA‐seq and ST at multiple developmental time points in skin samples from normal and hairless pigs. Using the data, we have: 1) identified multiple distinct epidermis and dermal related cell subtypes and provided a spatiotemporal transcriptomic atlas in pig fetus skin; 2) defined major cell lineage fate decisions in the process of epidermis and dermal morphogenesis development; 3) elaborated on the progenitor cells in Pc formation; 4) revealed the signaling pathways involved in the “first signal” that initiates Pc formation. Despite the high heterogeneity and asynchrony in skin development, our findings reveal the molecular pathways underlying epidermal and dermal formation, providing in‐depth insights into skin and associated HF development.

## 2 Results

**Figure 1:**
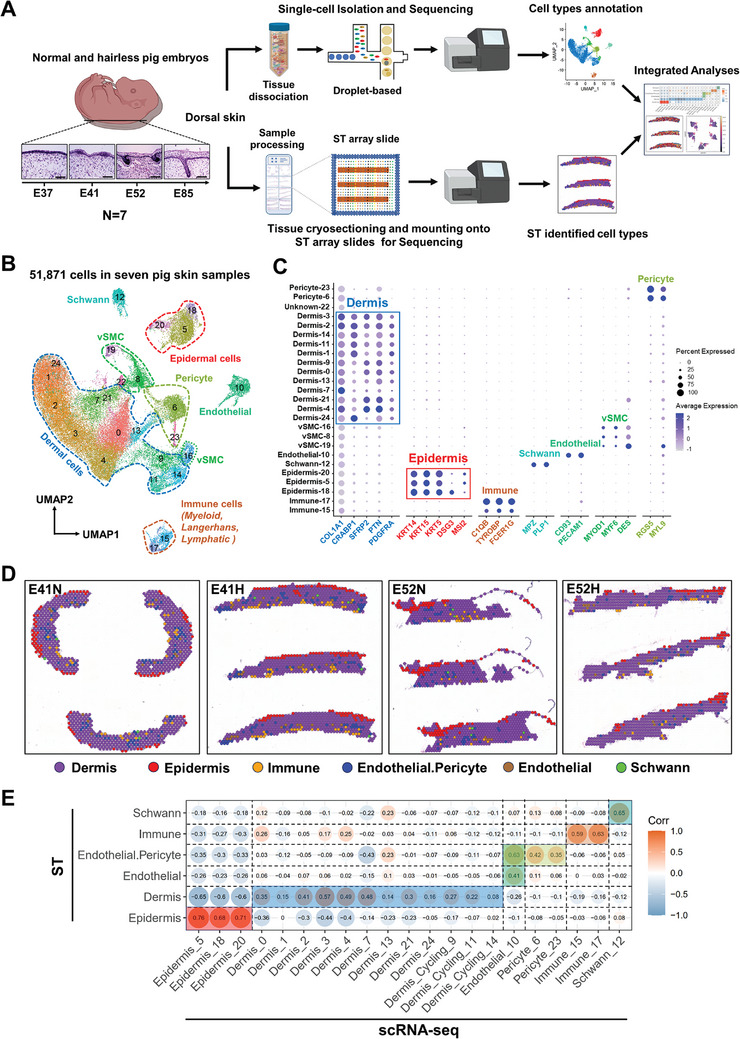
Single‐cell and spatial transcriptomic profiling of pig fetus skin. A) Schematic diagram showing the workflow for pig skin biopsy processing for single‐cell RNA sequencing (scRNA‐seq) and spatial transcriptomics (ST) analyses. Samples were obtained at four development timepoints, E37, E41, E52, and E85; B) UMAP plot revealing cellular heterogeneity with 25 distinct cell clusters of seven major cell types based on scRNA‐seq. The dot colors and numbers represent different cell clusters. vSMC = vascular smooth muscle cell; C) Bubble plot showing the representative marker genes for different cell clusters. The dot size reflects the percentage of each cluster expressing the gene, while the color saturation indicates the relative expression level; the epidermis and dermis are highlighted with red and blue solid line boxes, respectively; D) Spatial plots depicting the six major cell types identified by ST; E) Correlation heatmap between the common cell types identified by scRNA‐seq and ST. The vertical axis represents the six cell types of ST while the horizontal axis represents the major cell types of scRNA‐seq.

Skin biopsies were collected from seven pig fetuses at the following four time points: E37 (pre‐induction), E41 (induction), E52 (organogenesis), and E85 (cytodifferentiation). The biopsies were used for phenotype identification and ScRNA‐seq. The former revealed that three pigs were normal, three were hairless, and one was inconclusive (unknown) (Figure S1, Supporting Information). The scRNA‐seq data (**Figure** **1**A) generated from the biopsies revealed capture of 17143–18238 genes per sample (Table S1, Supporting Information). After quality control and integration (Figure S2A–C, Supporting Information), 51871 single cells across seven samples were retained for further analysis. Among the outputs generated by the Space Ranger (Table S2, Supporting Information), 8367 spots were deemed suitable for the subsequent integration analysis.

### 2.1 Single‐Cell and Spatial‐Temporal Transcriptomic Atlas of Pig Fetus Skin

To elucidate the cellular heterogeneity during skin morphogenesis, we first performed graph‐based clustering and annotated each cluster based on canonical cell‐type marker genes reported in human and mouse studies. We identified 25 cell clusters corresponding to seven major cell lineage branches with significantly enriched marker genes (Figure 1B,C; Figure S3, Table S3, and Data S1, Supporting Information). Of the seven cell types, the proportions of dermal and epidermal cell types were consistent at different time points in the normal and hairless pig groups, wherein the percentage of epidermal cells (the maximum proportion) first increased before E41 and then decreased, suggesting that epidermis development may undergo a major event at E41 (Figure S4A,B, Supporting Information). We verified the marker genes from humans and mice ^[^ ^27^ ^]^ indicative of the epidermis (*KRT14^+^* and *KRT15^+^*) and dermis (*COL1A1^+^* and *PTN^+^*) using immunofluorescence (IF) analysis (Figure S4C,D, Supporting Information). The single‐cell sequencing and IF results were highly consistent, confirming that the single‐cell data were accurately annotated.

To spatially map the cell types defined by our scRNA‐seq analysis, we used Visium ST, which generates whole‐genome expression data and retains spatial information. After data integration, graph‐based clustering, and cell‐type annotation based on canonical marker genes, six major cell types were identified in the ST (Figure S5A–D, Supporting Information). Figure 1D shows the spatial distribution of these ST‐generated cell types aligned with the skin organization and highlights clear demarcation of the dermis and epidermis. Various cell types, including immune cells, endothelial cells, pericytes, and Schwann cells, are dispersed within the dermal layer. The dermis exhibited the broadest distribution of skin tissues, highlighting its abundance (Figure 1D; Figure S5E, Supporting Information). This observation was consistent with the scRNA‐seq results, which revealed that the dermis accounted for the majority of cases (ca. 70%; Figure 1D; Figure S4A,B, Supporting Information). Furthermore, the seven cell types captured by scRNA‐seq were accurately mapped to their respective tissue locations in high‐resolution spatial plots (Figure S5F, Supporting Information; Figure 1D). This mapping revealed consistency in cell type annotation between scRNA‐seq and ST data. Additionally, correlation analyses demonstrated high concordance between the transcriptomes of common cell types identified by both scRNA‐seq and ST (Figure 1E).

### 2.2 Pig Epidermis is a Better Representative of Human Epidermis Than Mouse Epidermis

**Figure 2:**
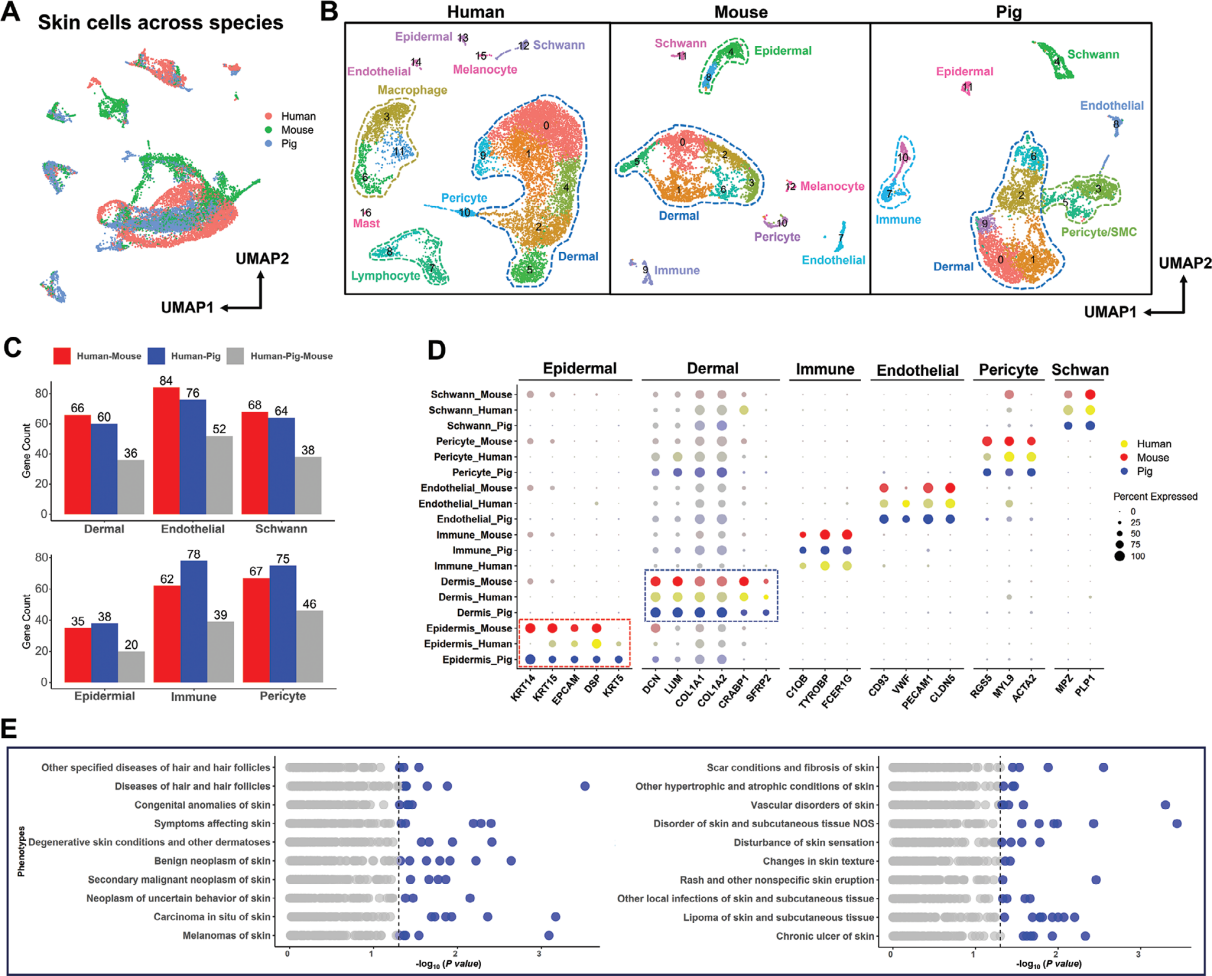
Cross‐species cell atlas of humans, mice, and pigs. A) UMAP visualization of integrated scRNA‐seq data from humans, mice, and pigs; B) UMAP plot revealing cellular heterogeneity with major cell types in the skin tissues of humans, mice, and pigs. Different colors represent different cell clusters; C) Bar charts showing the number of conserved cell‐type marker genes between humans and mice (red), humans and pigs (blue), and among three species collectively (grey); D) The species‐split bubble plot showing cross‐species comparison of selected top markers for all major cell types profiled across all species. The dot size reflects the percentage of each cell type expressing the gene, while the color saturation represents the relative expression level. The color scheme is as follows: yellow for humans, red for mice, and blue for pigs. The epidermis and dermis cells are boxed with red and blue, respectively; E) Phenome‐wide association study (PheWAS) revealed that the conserved marker genes in the skin of pigs and humans are strongly involved in human skin‐related diseases.

Figure 1 shows highly heterogeneous skin cells, indicating that multiple cell types are involved in skin development. To support our pig skin results, we integrated publicly available scRNA‐seq data from humans and mice to compare cell types across species (**Figure** **2**A). Independent graph‐based clustering for each species revealed that humans, mice, and pigs shared conservation in the main skin cell types, including dermis, epidermis, endothelial cells, Schwann cells, pericytes, and immune cells (Figure 2B). Plotting these cell types in the integrated uniform manifold approximation and projection (UMAP) yielded large overlapping distributions of identical cell types across the three species, indicating the conservation of gene expression across different organisms (Figure 2A; Figure S6, Supporting Information). Moreover, greater conservation of cell‐type‐specific marker genes has been observed between mice and humans in dermal, endothelial, and Schwann cells. Conversely, pigs exhibited more conserved cell type‐specific marker genes in the epidermis, immune system, and pericytes (Figure 2C; Data S2, Supporting Information). This supports the findings of previous studies indicating that the pig epidermis is more representative of the human epidermis than the mouse epidermis.^[^ ^28^ ^]^ Figure 2D shows the classical marker genes for skin cell types, allowing comparisons across species. Marker genes in the epidermis (*KRT15+* and *EPCAM+*) and dermis (*DCN+* and *COL1A1+*) showed consistent expression patterns across all three species. Conversely, certain marker genes such as *KRT14+* were detected only in certain species and marked epithelial cells (mouse and pig), *KRT5+* marked epithelial cells (human and pig), and *VWF+* marked endothelial cells (human and pig). As for pig‐human conserved cell‐type‐specific genes, PheGWAS explorations reflected that these genes were strongly associated with human skin‐related diseases (Figure 2E). Overall, compared with the other animal models examined here, porcine skin was found to better represent human skin, suggesting that porcine skin models would more accurately represent human skin for studying skin‐related complex diseases.

### 2.3 Epidermis Cell Lineages during Skin Development

**Figure 3:**
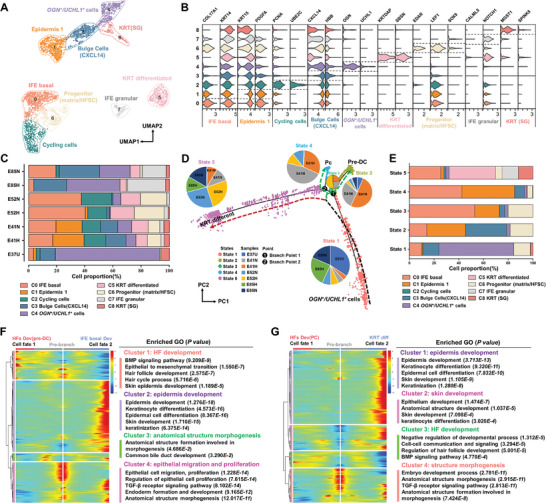
Recapitulating epidermis cell fate decision during skin development. A) UMAP plot of the nine cell subtypes in the epidermis; B) Violin plots of representative marker genes for annotating the nine cell subtypes in the epidermis. The y‐axis represents the nine cell subtypes of the epidermis while the x‐axis represents the characteristic marker genes for the different cell subtypes; C) Proportions of the nine cell subtypes in the epidermis of different normal samples; D) Pseudo‐time ordering analysis revealing all epidermis lineage cells as two branches and five states. The color dashed lines represent different evolutionary tracks, in which the black one represents pre‐branch while red and green lines represent epidermis and hair follicle (HF) development, respectively; Each dot represents one cell, and five colors of pink, khaki, green, blue, and violet represent cell states 1, 2, 3, 4 and 5, respectively; the pie charts show the proportions of cells from the seven samples at each cell state; E) Bar charts showing the proportions of the nine cell subtypes of epidermis at each of the five cell states; F) Heatmap illustrating DEG (differentially expressed genes) dynamics during the cell fates of HF development (pre‐DC) and IFE basal development; the DEGs are clustered into four gene sets by Gene Ontology (GO) enrichment analysis (right panel); G) Heatmap illustrating DEG dynamics toward HF development (Pc) and KRT differentiation fate along the pseudo time; The DEGs are clustered into four gene sets based on GO enrichment analysis; Pc: hair placode; pre‐DC: pre dermal condensate; IFE: interfollicular epidermis; KRT: keratinocyte; SG: sebaceous gland; HFSC: hair follicle stem cells; KRT diff: keratinocyte differentiation.

Epidermal cell lineages (clusters 5, 18, and 20 in Figure 1B) were extracted for sub‐clustering. All nine subtypes were annotated based on the expression of canonical cell type markers (**Figure** **3**A,B **;** Table S4 and Data S3, Supporting Information). Figure 3C shows the proportions of the nine cell subtypes in the epidermis of each sample. Notably, *OGN^+^/UCHL1^+^* cells mainly existed at E37U, whereas the proportion of interfollicular epidermis (IFE) basal and progenitor interfollicular epidermis (IFE) cells increased from E41 to E52. No significant differences were observed in the proportions of the distinct epidermal subtypes between the normal and hairless groups (Figure S7A, Supporting Information). However, differentially expressed genes (DEGs) were detected in clusters 0 and 1 (Figure S7B, Supporting Information). These DEGs are associated with the regulation of epidermal layer development (Figure S7C, Supporting Information).

Pseudotime trajectory analyses divided epidermal cells into five developmental states on two cell lineage branches (Figure 3D). The pseudotemporal ordering of cells also reflected the developmental time points at which the cells were sampled (Figure S7D, Supporting Information). E37 was primarily located at the initial position of state 1, E85 (N & H) at state 5, and E41 (N & H) and E52 (N & H) in the midsection of the trajectory (Figure 3D pie plots and Figure S7E, Supporting Information). In addition, state 1 was mainly composed of *OGN* ^+^/*UCHL1* ^+^ cells (Cluster 4), while state 5 mainly comprised differentiated KRT‐ and IFE‐related cells (Clusters 0, 5, 7, and 8) in both normal and hairless groups (Figure 3E). Focusing on branch point 1, representing cell differentiation from *OGN* ^+^/*UCHL1* ^+^ cells to pre‐DC and IFE basal cells (states 2 and 3) (Figure 3F), we determined the genes that were significantly differentially expressed as a function of the pseudotemporal ordering of cells on either side of this branch point. Based on the pattern of differential expression observed for these genes, we identified four gene groups with highly consistent gene expression dynamics along the cell differentiation pathway between *OGN* ^+^/*UCHL1* ^+^ and pre‐DC cells. These expression patterns suggest that *OGN* ^+^/*UCHL1* ^+^ cells represent the early progenitors of skin development. Similar to the analysis performed for branch 1, the results indicated that branch point 2 was related to mature Pc development and HF morphogenesis (state 4), while KRT differentiation (state 5) occurred along the alternating cell fate, indicating a common progenitor (Figure 3G).

**Figure 4:**
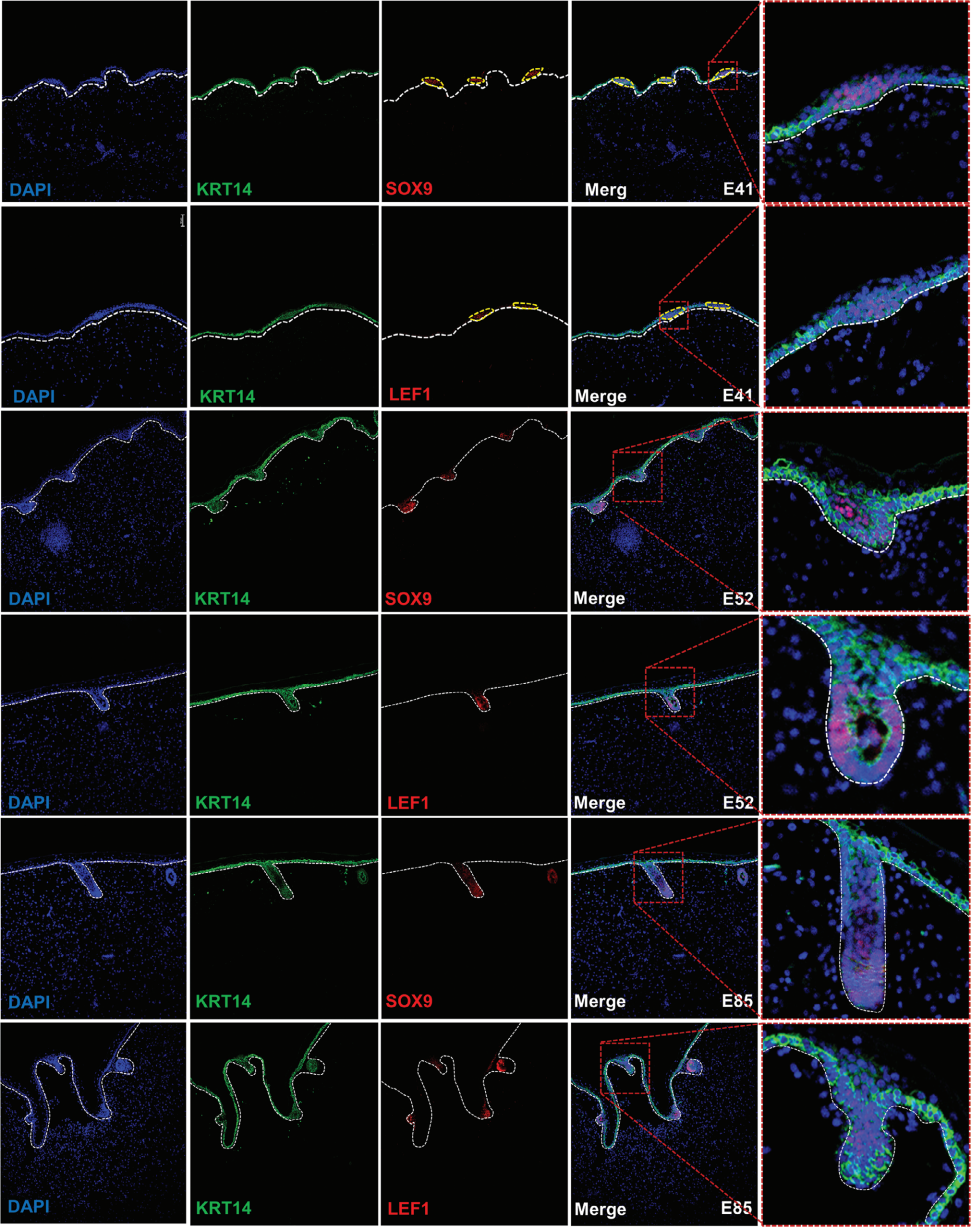
Visualization of classical HF markers expression. Immunofluorescence (IF) analysis of SOX9 and LEF1 expression in developmental skin tissues at different time points during hair follicle morphogenesis. The epidermis was detected using *KRT14*. Dotted lines in the IF images represent the dermal and epidermal boundaries. Scale bars, 50 µm.

Finally, IF verification was performed at different stages of HF morphogenesis using classical marker genes. The results showed that LEF1 and SOX9 were spatially co‐expressed with the HF marker genes, consistent with the scRNA and ST results (**Figure** **4**).

Taken together, we successfully sub‐classified nine epidermal cell subtypes and recapitulated two lineage differentiation trajectories that describe the gene expression changes that occur during epidermal development and HF morphogenesis.

### 2.4 Dermis Cell Lineage during Skin Development

**Figure 5:**
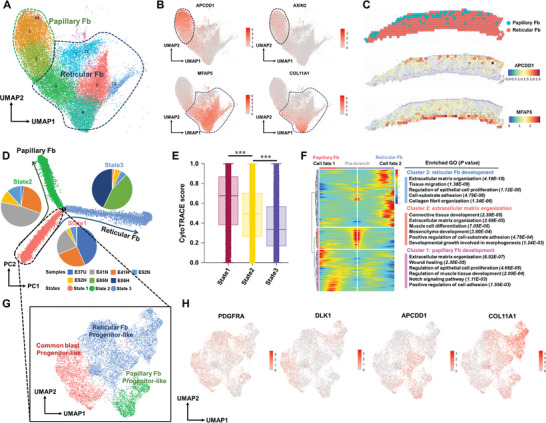
The heterogeneous origins of dermal fibroblasts. A) UMAP plot showing the diverse fibroblast subtypes of dermal cells; B) Expression distribution of marker genes by annotated fibroblast subtypes; C) Spatial plot depicting fibroblast subtypes from single‐cell RNA sequencing using cell2location; Spatial feature plots showing the expression of marker genes by the annotated cell types; D) Pseudo‐time trajectory revealing all dermis lineage cells as two branches and three states. The solid lines with arrows represent different evolutionary tracks, including papillary and reticular fibroblast development; each dot represents one cell, and is colored by states; the pie charts show the proportions of cells from the seven samples at each cell state; E) Statistical results showing CytoTRACE analysis of dermal cells at different states. Kruskal‐Wallis nonparametric analysis followed by Duncan's multiple comparison tests was used to calculate the statistical significance. ^***^ *p* < 0.001; F) Heatmap illustrating DEG (differentially expressed genes) dynamics toward the fates of papillary and reticular fibroblast development; the DEGs are clustered into three gene sets by Gene Ontology enrichment analysis (right panel); G) Subclustering of state 1 cells in the trajectory of dermis lineage cells, color labeled by cell subtypes; H) Expression levels of diverse marker genes by annotated cell subtypes.

Two major subtypes of dermal fibroblasts were identified primarily using cluster‐specific marker genes (**Figure** **5**A,B): papillary fibroblasts (*APCDD1* and *AXIN2*) and reticular fibroblasts (*MFAP5* and *COL11A1*) (Table S5, Supporting Information). The physical localizations of the dermis subtypes in the tissues were consistent with their anatomical locations identified histologically; compared to the reticular dermis, the papillary dermis is the superficial layer of the dermis that lies immediately below the epidermis^[^ ^29^ ^]^ (Figures 5C and 1D). The representative expression features of these dermal fibroblast subtypes are illustrated by ST (Figure 5C). No significant differences were observed in the proportions of the various clusters of dermal fibroblasts between the normal and hairless groups (Figure S8A, Supporting Information). However, notable variations were detected in the number of DEGs among the different cell clusters, particularly in clusters 1, 2, 0, and 3 (Figure S8B, Supporting Information). These DEGs were associated with the extracellular matrix and extracellular structures of the dermal layer (Figure S8C, Supporting Information).

Using Monocle2 and CytoTRACE, pseudo‐time trajectory analyses divided dermal fibroblasts into three states, with two distinct cell fates arising from state 1 (Figure 5D,E; Figure S8D, Supporting Information). Based on the gene expression profiles along the trajectory, the fibroblasts were closely related to “extracellular matrix organization,” “cell‐substrate adhesion,” and “connective tissue development,” reflecting the role of dermal fibroblasts in generating the extracellular matrix and forming the connective tissue that maintains skin morphology and homeostasis (Figure 5F). Fibroblast heterogeneity can be considered based on the stage of development, tissue of origin, or tissue microenvironment. In mice, dermal fibroblasts are derived from common fibroblast progenitor cells and differentiate into specific lineages by postnatal day two (P2).^[^ ^30^ ^]^ To investigate the origin of dermal fibroblast heterogeneity at the embryonic stage, the cells in state 1 were isolated at E37 for sub‐clustering (Figure 5D pie plots; Figure S8E, Supporting Information). Based on previously reported markers (Table S5, Supporting Information) of papillary fibroblasts (*APCDD1*), reticular fibroblasts (*COL11A1*), and fibroblast progenitors, the cell identity of dermal fibroblast origin was determined (Figure 5G,H).

### 2.5 Progenitor Origins of Pc in Early HF Development

**Figure 6:**
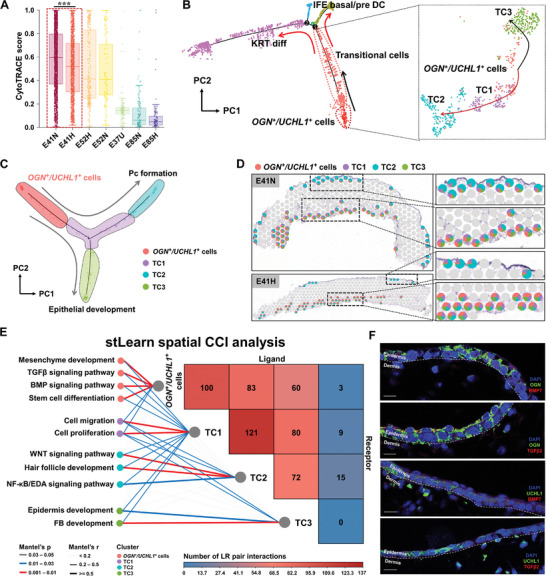
Molecular mechanism and transcriptional regulatory networks in hair placode formation. A) Cell stemness determined by CytoTRACE analysis of the epidermal cells of different samples. Kruskal‐Wallis nonparametric analysis with Duncan's multiple comparison tests was used to calculate the statistical significance. ^***^ *p* < 0.001; B) Cells in state 1 were classified into four clusters, *OGN* ^+^/*UCHL1* ^+^ cells and transitional cells 1, 2, 3. TC: transitional cell; C) Remodeling of trajectory development before placode formation; D) The proportions of *OGN* ^+^/*UCHL1* ^+^ cells and TC1/2/3 in each spot were detected in normal (upper panel) and hairless (lower panel) fetuses at E41; E) Cell‐cell interaction analysis of ligands and receptors in the four clusters, *OGN* ^+^/*UCHL1* ^+^ cells and transitional cells 1, 2, 3 (TC1, TC2, and TC3). The number of overlapped ligand‐receptors is displayed on the triangle heatmap. The lines illustrate the associated biology processions (BPs) in each cluster; the color and thickness of the lines denote the significance and correlation of the association (Mantel's *P* and Mantel's r), respectively; F) Immunofluorescence (IF) validation of OGN and UCHL1 co‐localization with BMP7 and TGFβ2 in the epidermis of E37 fetal pig skins. The dotted line represents the boundary between the epidermis and dermis; Scale bar = 10 µm.

In normal pigs, Pc formation occurs from E37 (pre‐induction) to E41 (induction); in contrast, hairless pigs lack Pc cells (Figure S1, Supporting Information). We hypothesized that Pc formation is aberrant in hairless pigs. To test this hypothesis, we first applied CytoTRACE^[^ ^31^ ^]^ to evaluate the stemness scores of epidermal cells. The findings revealed a decline in stemness from E41 to E85, while the lower stemness in E37U could be attributed to its distinct cell type composition compared to the other samples (**Figures** **6**A and 3C). In particular, the proportion of HF stem cells (HFSCs) with high stemness^[^ ^32^ ^]^ and gene expression‐active cycling cells^[^ ^32a^ ^]^ increased from 0 to nearly 20% between E37 and E41, and was maintained at E52, resulting in lower stemness at E37 than at E41 and E52. When comparing the normal and hairless samples, a noteworthy disparity in stemness was observed only at E41 (*P* = 4.96e‐08) (Figure 6A). These results support our hypothesis that the hairless phenotype occurs between E37 and E41 during HF morphogenesis (particularly state 1 cells).

We isolated all the cells within state 1 for fine‐grained clustering and pseudo‐time analysis. This indicated that *OGN* ^+^/*UCHL1* ^+^ cells formed two differentiation branches through TC1 toward TC2 (Pc formation) and TC3 (epithelial fate) (Figure 6B,C). Differential expression analysis between the two branches (*OGN* ^+^/*UCHL1* ^+^ →TC1→TC2 and *OGN* ^+^/*UCHL1* ^+^→TC1→TC3) revealed that TGFβ and BMP signaling were enriched at the cell fate decision point, indicating the importance of these signaling pathways for regulating HF morphogenesis (Figure S9A, Supporting Information). Using Cell2location,^[^ ^33^ ^]^ the spatial localization of *OGN* ^+^/*UCHL1* ^+^ cells was determined for the first time at E37 (Figure S9B, Supporting Information). Additionally, our investigation revealed activated *BMP* and *TGFβ* signaling pathways within this specific region (Figure S9C, Supporting Information). This finding highlights the significance of *OGN* ^+^/*UCHL1* ^+^ cells and the involvement of critical signaling pathways in spatial patterning. No differences in cell proportions were apparent between normal and hairless pigs for *OGN^+^/UCHL1^+^* cells and TC1 at E41 (Figure 6D). Although Pc (TC2) cells were detected in the epidermis at E41 of normal samples but were greatly reduced in E41 hairless samples (Figure 6D right panel).

Next, we sought to describe the mechanisms of cell signaling that facilitate *OGN* ^+^/*UCHL1* ^+^ to Pc cell transition by ligand‐receptor (LR) cell‐cell interaction analysis. Several more significant LR interactions were observed between *OGN* ^+^/*UCHL1* ^+^ and TC1/2 cells than between *OGN* ^+^/*UCHL1* ^+^ and TC3 cells. TC1 strongly interacted with both *OGN* ^+^/*UCHL1* ^+^ cells and TC2 (r > 0.2, *p* < 0.03), whereas weak correlations were found between TC3 and other cell subtypes (r < 0.2, *p* < 0.05) (Figure 6E).

In addition, the transcriptional regulatory networks in the early stage of Pc formation (Figure S10A,B, Supporting Information) show that the LR interactions engaged by *OGN* ^+^/*UCHL1* ^+^ cells were enriched in the biological process “stem cell differentiation,” *BMP* and *TGFβ* signaling pathways (Figure S10A, Supporting Information, left). In TC1, the LR pairs were enriched for “cell proliferation” and “cell migration,” but these were only observed in normal samples and not in hairless pigs, suggesting the cellular behavior caused by differential cell‐cell signaling could underlie the reduced Pc formation in hairless pigs (Figure S10A, Supporting Information, middle). As for TC2, significant LR interactions were enriched for classical HF signaling pathways such as the *WNT* and *NF‐kB/EDA* pathways (Figure S10A, Supporting Information, right). Furthermore, we located the *OGN^+^/UCHL1^+^* cells in E37 epidermal tissues using their marker genes along with representative genes from the BMP and TGFβ signaling pathways (Figure 6F; Figure S9D,E, Supporting Information). This confirmed the existence of *OGN* ^+^/*UCHL1* ^+^ in our spatial samples and further supported the role of BMP and TGFβ signals in early HF development.

Overall, our results revealed two differentiation trajectories, one from *OGN* ^+^/*UCHL1* ^+^ cells→TC1→TC2 leading to normal Pc formation, the other from *OGN* ^+^/*UCHL1* ^+^ cells→TC1→TC3 leading to epithelial development. Closer examination suggested that *OGN* ^+^/*UCHL1* ^+^ cell abnormalities in proliferation and migration lead to abnormal Pc formation in hairless pigs and that BMP and TGFβ are the first signaling pathways that trigger *OGN* ^+^/*UCHL1* ^+^ cells to undergo Pc formation.

## 3 Discussion

Skin morphogenesis is a complex process involving the interaction of multiple cell lineages derived from the epidermis and dermis, and is especially apparent at the boundary between the epidermis and dermis.^[^ ^27^, ^34^ ^]^ Although dermal and epithelial cell fate decisions during embryonic skin development had been reported in humans,^[^ ^35^ ^]^ mice,^[^ ^19b^ ^]^ and sheep^[^ ^36^ ^]^ based on scRNA‐seq data, in this study, we used a high‐throughput scRNA‐seq and high‐spatial‐resolution ST framework to generate an integrated spatiotemporal transcriptome atlas of skin morphogenesis in pig fetuses and validated the cell types using immunofluorescence. Comparing the skin cell lineages of pigs with those of humans and mice (Figure 2) revealed that the pig epidermis is a better representative of the human epidermis than mice and that the overlapping genes between pigs and humans are strongly associated with human skin‐related diseases as reflected by PheGWAS analysis findings. This suggests that our pig model is a valuable resource for understanding human skin development and is, therefore, more relevant from a clinical perspective, such as hair‐loss conditions in adults (e.g., alopecia).

Compared to scRNA‐seq, the number of detected cell types in ST was lower (6 vs 7), including a mixed type labeled as “Endothelial.Pericyte”. This discrepancy may be attributed to the lower resolution of ST, which typically covers 5‒100 cells per spot.^[^ ^20b^ ^]^ By combining the high resolution of scRNA‐seq and spatial information of ST with state‐of‐the‐art bioinformatics methods, we described cellular‐ and tissue‐level molecular developmental events in HF morphogenesis. In particular, based on the gene expression patterns of each cell state in the pseudo‐time analysis and scRNA‐seq/ST data integration, we summarized the differentiation relationship of each cell subtype during HF development and how these cells co‐occur spatially in the epidermis. This confirmed previous findings that the temporal dynamics of cell subtypes during HF morphogenesis are consistent with their morphology.^[^ ^7^ ^]^ Overall, the combination of scRNA‐seq and ST provided a comprehensive characterization of HF cell types in their native environments, which could not have been determined using either modality alone.

Researchers have primarily used Monocle2 to construct pseudotemporal trajectories to investigate dermal and epidermal cell fate determination during early skin development. Due to differences in sample collection and the stage of investigation, the developmental trajectories constructed by different studies may also diverge. For instance, Ge et al. built a trajectory of dermal development that encompassed a branch of dermal adipocytes^[^ ^19b^ ^]^ which could be attributed to the capture of subcutaneous adipose tissue during sample processing. Their trajectories also included dermal cell types associated with HF development. Conversely, our trajectory identified two primary types of dermal fibroblasts and annotated three subtypes of fibroblast progenitor‐like cells during the early stages of dermal development. This discrepancy could be attributed to the notable thickness of the porcine dermal layer, which results in a relatively limited proportion of dermal cell types associated with HFs. Previous studies have explored and reconstructed two differentiation trajectories in mature HFs.^[^ ^37^ ^]^ Here, we identified two differentiation trajectories during early skin development. Notably, Ge et al.’s epidermal trajectory aligns with our observations, pointing toward cell fates relevant to the epidermis and HFs.^[^ ^19b^ ^]^ Within their trajectory, the cell fate branches of the matrix, inner root sheath (IRS),^[^ ^19b^ ^]^ and hair shaft corresponded to the subsequent developmental progression of Pc cells. This suggests a process whereby Pc cells transition from an induction marker to HFs with mature structures. Focusing on this process, Ritsuko et al. conducted a high‐resolution study combining scRNA‐seq and lineage tracing after the induction stage.^[^ ^38^ ^]^ They revealed that the fate of Pc cells was governed by their positions within the Pc. For example, the outer ring of the Pc forms a bulging region in HFSCs. In our study, we investigated the differentiation of Pc cell, with an additional focus on the pre‐induction stage. Furthermore, we identified a crucial subpopulation of progenitor‐like cells that played a pivotal role in the origin of Pc cells, furthering the understanding of Pc cell fate determination and HF morphogenesis.

Previous studies have shown that the occurrence of hairless traits in pigs is related to the blocking of Pc formation.^[^ ^7^, ^39^ ^]^ However, the events that lead to Pc formation remain sparsely delineated,^[^ ^27^, ^40^ ^]^ because conventional tools cannot isolate Pc from the embryo skin. With the high sensitivity and resolution achieved by the combined use of scRNA‐seq and ST, we elucidated that Pc formation is preceded by an *OGN* ^+^/*UCHL1* ^+^ cell population. This is in contrast to previous studies, which implicated the WNT signaling pathway as the first essential signal for Pc formation.^[^ ^41^ ^]^ Other studies also indicated that the BMP and TGFβ signaling pathways are critical for HF morphogenesis,^[^ ^27^, ^34^ ^]^ with BMP signaling believed to modulate or reinforce the quiescence of HF stem cells upon self‐renewal and play an important role in cell identity maintenance during HF morphogenesis.^[^ ^42^ ^]^ TGFβ‐related genes are involved in the pathways that control the hair growth phase transition and promote hair growth by preventing catagen progression.^[^ ^16^, ^43^ ^]^ Ours is the first study to illustrate that BMP and TGFβ signaling pathways play a key role in early Pc morphogenesis formation, and occurs prior to the WNT signaling pathway. However, the detailed mechanism needs further investigation.

We also observed that *OGN* ^+^/*UCHL1* ^+^ cells may be impaired in hairless pigs compared to normal pigs. In the cell fate commitment (*OGN* ^+^/*UCHL1* ^+^ cells→TC1→TC2) for Pc formation, we found no difference in *OGN* ^+^/*UCHL1* ^+^ cells in normal and hairless fetuses, but after being transmitted to TC1, the expression and proliferation of TC1 reduced in hairless pigs, which led to a decrease of TC2, thereby mitigating the suppression of Pc formation.

Despite our study providing detailed insights into the single‐cell and spatial transcriptomes during fetal pig skin development, further investigation is needed to understand the transcriptional changes in subsequent developmental stages and adult skin. Moreover, our study had a relatively small sample size, and although we made efforts to include samples from different developmental periods, there might still be some sample bias. Additionally, while we extensively investigated cell types and subtypes, there is a possibility of unidentified cell subtypes or novel cell types that require further exploration.

## 4 Conclusion

In this study, we generated single‐cell and spatial transcriptomes of fetal pig skin during different periods of development. After systematically investigating the presence and plasticity of the cell types and subtypes, together with their respective gene expression programs and spatial positions, we created an integrated transcriptome atlas of the entire embryonic skin origin and captured a molecular snapshot of HF progenitors and their niches. Our data revealed the precursor cell states and molecular events during the earliest phase of skin and HF morphogenesis. To our knowledge, this is the first study to characterize single‐cell and spatial gene expression during pig skin development at a spatiotemporal resolution. Moreover, we used the data to delineate molecular and cellular events that precede the emergence of Pc morphogenesis and discovered that *OGN* ^+^/*UCHL1* ^+^ cells constitute the progenitor cell population at the earliest phase of Pc formation, thereby revealing that BMP and TGFβ signaling pathways likely initiate the development of Pc and hence HF morphogenesis.

## 5 Experimental Section

### Ethics

All animal procedures were evaluated and authorized by the Institutional Animal Care and Use Committee (IACUC) of China Agricultural University. Samples were collected in strict accordance with the protocol approved by the IACUC (permit number DK996).

### Samples for scRNA‐Seq and ST Experiments

The pig hairlessness, presenting as a congenital absence of hair, was previously characterized as an autosomal non‐sex‐linked inheritance in the established population. This was attributed to the genetic defects that affect Pc formation during embryonic development.^[^ ^39^ ^]^ Using four pairs of normal boars and hairless sows from a population of Yorkshire pigs, mating experiments were performed to generate four litters of porcine fetuses at different embryonic time points. Skin biopsies of seven pig fetuses were collected at four time points: E37 (pre‐induction), E41 (induction), E52 (organogenesis), and E85 (cytodifferentiation). The association between specific time points of embryonic development and the stages of HF morphogenesis was deduced from a previous study.^[^ ^7^ ^]^ The number of HFs was identified via H&E staining to classify each fetus as hairless (< 1 HF/cm^2^) or normal (> 4 HF/cm^2^). A fetal sample was obtained at embryonic time point E37, which corresponds to the stage before HF formation. The phenotype of this sample could not be identified because of the absence of the characteristic Pc marker at E37, resulting in its designation of E37U. One pair of hairless and normal samples from the same litter was collected at three other time points (E41, E52, and E85) and referred to as E41H, E41N, E52H, E52N, E85H, and E85N to represent the hairless and normal fetuses in E41, E52, and E85, respectively. Each of the seven samples was equally divided for single‐cell RNA sequencing and spatial transcriptome analyses.

### Single‐Cell RNA Sequencing

*Preparation of Single‐Cell Suspensions*: To prepare single‐cell suspensions from dorsal skin tissues, fetal back skin tissues were finely minced with a scalpel and enzymatically digested using a 0.25% trypsin/EDTA solution at 37 °C for 5 min. Subsequently, the obtained skin tissues were incubated with 2 mg mL^−1^ type IV collagenase and 20 U µL^−1^ DNase I at 37 °C for 40 min. Single‐cell suspensions were obtained by mechanically dissociating the skin tissues through repeated aspiration and dispensing with a pipette ten times. To ensure the removal of clumps or debris, the resulting cell suspensions were filtered using a 100 µm and a 40 µm nylon cell strainer (BD Biosciences, San Jose, CA, USA). The flow‐through was spun down and resuspended in erythrocyte lysate and 37% Percoll to remove the erythrocytes. The settled cells were resuspended in PBS (phosphate buffered solution) containing 0.5% (w/v) BSA (bovine serum albumin). Cell suspensions were passed through a cell strainer, and 7‐Aminoactinomycin D (Molecular probes by Life Technologies) was added for dead cell identification. Live total skin cells were sorted using a BD Influx Cell Sorter (BD Biosciences) equipped with a 100 mm nozzle. Data were analyzed using the FlowJo v10.0.8r1 software (FlowJo LLC, BD).

*Single‐Cell Library Preparation and Sequencing*: The single‐cell suspensions were subjected to library preparation using the Chromium Single Cell 3′ Library and Gel Bead Kit v2 (10x Genomics, Pleasanton, CA, USA), following the manufacturer's instructions. Barcoded cDNA libraries were constructed using the 10x Genomics Chromium barcoding system. The libraries were sequenced on a HiSeq X Ten platform (Illumina, San Diego, CA, USA), and 150 bp pair‐ended reads were generated for downstream analysis.

*Raw Data Processing and Quality Control*: The raw sequencing data of each sample were processed and aligned to the Sscrofa11.1 reference genome using the Cell Ranger pipeline (release 4.1.0). The per‐sample data were normalized by down‐sampling the reads across the samples, as suggested by Cell Ranger. Next, a raw unique molecular identifier (UMI) count matrix was generated that was used as the input for the R package Seurat^[^ ^44^ ^]^ (version: 4.0.1) to establish the Seurat object. Cells with fewer than 200 features or >20% of mitochondrial genes were excluded. The doublets were also removed by removing the cells identified by DoubletFinder^[^ ^45^ ^]^ (version: 2.0.3). Genes that were expressed in fewer than three cells were excluded. Following quality control, 51871 cells were retained.

*Identification and Annotation of Clusters*: Following quality control, cell normalization and regression were performed based on the expression matrix using Seurat to obtain scaled data. The Harmony package was used to remove potential batch effects using the default parameters. A graph‐based cluster method based on the top 10 principal components estimated from 2000 genes was conducted with highly variable expression. A resolution of 0.8 was selected, and then clustered the cells on a 2D UMAP plot. To detect genes with enriched expression in each cluster, the Seurat function FindAllMarkers was performed using a nonparametric Wilcoxon rank‐sum test with *P* values adjusted by Bonferroni correction. Significance was set at *p* < 0.05. For sub‐clustering analyses, the corresponding clusters were extracted and reclassified using the above‐described method. A series of well‐recognized cell‐type markers were used to assign cell‐type identities (Tables S3–S5, Supporting Information).

*Trajectory Analysis and CytoTRACE*: Monocle2^[^ ^46^ ^]^ was implemented to acquire the pseudo‐time differentiation trajectory of embryonic skin, in which UMI count data were used as input. Seurat identified variable genes as ordering genes for constructing the trajectory of the cells. After the trajectory construction, the cells in each state were mapped back onto the original object. CytoTRACE^[^ ^31^ ^]^ was used to predict the differentiation potential of the cells. Cells with higher CytoTRACE scores represented higher stemness (less differentiation) within the given dataset, and vice versa.

*Cross‐Species Comparison*: To compare developing skin across species, a normal pig sample was selected at E85. Publicly available scRNA‐seq datasets from human and mouse embryonic skin samples were also downloaded. The human skin dataset was derived from healthy fetal skin^[^ ^47^ ^]^ (https://developmental.cellatlas.io/diseased‐skin), whereas the mouse skin data were obtained from embryonic E16.5^[^ ^19b^ ^]^ (accession number: GSE131498). Graph‐based clustering and cell‐type annotation were first conducted and cell‐type‐specific marker genes were examined separately for each species. To evaluate the similarity between these species, the overlapping genes of the top 200 cell type‐specific marker genes after homologous conversion were identified as conserved genes across species. Next, we converted the genes of the datasets (pig and mouse) to homologous human genes, followed by CCA^[^ ^48^ ^]^ integration based on the intersection of homologous genes. The integrated dataset was embedded and visualized using UMAP and labeled according to annotations in the separately processed datasets.

*Evaluation of Key Genes of Skin and HF Traits with Human Data Sources*: To evaluate whether the key genes detected in skin development and HF traits in pigs were associated with similar phenotypes in humans, ExPheWas,^[^ ^49^ ^]^ a PheWAS browser and platform based on the GWAS atlas, was used to identify the related genes of each phenotype. The study focused on the genes involved in critical pathways of Pc formation; the genes with *P* values < 0.05 were significant.

*Differential Gene Expression Analysis*: The Seurat function FindMarkers with the Wilcoxon rank‐sum test algorithm was used under the following criteria to identify DEGs between normal and hairless groups: log_2_FC > 0.25, adjusted *P* value < 0.05.

*Gene Enrichment Analysis*: Gene Ontology (GO) term analysis and Kyoto Encyclopedia of Genes and Genomes (KEGG) pathway enrichment analysis were performed using ClusterProfiler (version: 4.0.0). GO terms and pathways were enriched in the human database because of the lack of studies in pigs. The Benjamini–Hochberg (BH) method was used for multiple testing.

### Spatial Transcriptomics

*Tissue Harvesting*: Visium spatial transcriptomic analysis was performed on the seven biopsies used for scRNA‐seq. Whole skin samples were isolated using an aseptic technique and placed in ice‐cold sterile Hank's Balanced Salt Solution. Fresh tissues were immediately embedded in optimal cutting temperature compound (OCT) blocks and frozen in a liquid‐nitrogen‐cooled isopentane bath.

*Library Preparation and Sequencing*: OCT blocks were sectioned at 10 µm thickness, 6.5 mm × 6.5 mm in size, and mounted for four stages (three sections from each sample), with one stage per capture area on a Visium slide. The sections were then stained with H&E following the 10x Genomics Visium fresh‐frozen tissue processing protocol. Next, the sections were imaged using a Zeiss PALM MicroBeam laser capture microdissection system, and libraries were generated following the 10x Genomics protocol. The optimal permeabilization time for 10 µm thick pigskin sections was 12 min. The samples were sequenced on an Illumina NovaSeq 6000 platform.

*Raw Sequencing Data Processing and Quality Control*: The sequencing output of each sample was processed and aligned to the Sscrofa 11.1 reference genome using Space Ranger software (version 1.2.2) from 10x Genomics, with UMI counts summarized for each spot. To distinguish tissue‐overlaying spots from the background, the spots were detected and retained according to the histological images. Only the barcodes associated with these spots were used to generate the filtered UMI count matrices. Low‐quality spots with gene counts < 200 were filtered out.

*Identification and Annotation of Spot Clusters*: Following quality control, Seurat^[^ ^44^ ^]^ was used to process ST data for subsequent analysis. ST samples were normalized independently by running the SCTransform function with default parameters, with the top 3000 genes with most variable expression selected for downstream anchor‐based integration and cluster analysis. The top 20 PCs with a resolution of 0.8 were used to identify spatial clusters by running the FindNeighbours and FindClusters functions. The spot clusters were manually annotated based on cluster‐specific marker genes identified using the FindAllMarkers function with default parameters, including the Wilcoxon rank sum test with *P* values adjusted by Bonferroni correction. The significance level of the adjusted *P* value was set to 0.05.

*Integration of scRNA‐seq and ST*: The scRNA‐seq dataset was used as a reference and employed anchor‐based integration using the Seurat package to achieve spot‐based enrichment for each reference cell type. Cell‐type prediction scores were calculated for each spot and subsequently visualized using the SpatialFeaturePlot function. Correlation analysis was conducted to assess the similarity of common cell types between scRNA‐seq and ST data. Seurat‐scale data of significant marker genes that exhibited overlap between the two datasets were employed for this analysis. The Pearson correlation coefficient was used as a statistical measure to evaluate the relationship among cell types. The cell‐type composition of each spot in Visium ST was inferred by the Cell2location, which was a Bayesian model that uses a reference cell type.^[^ ^50^ ^]^ The expression signatures of the cell types were calculated using the scRNA‐seq dataset, mapped the learned cell‐type signatures onto slides and visualized them. The default parameters were used to train Cell2location model.^[^ ^33^ ^]^

*Cell–Cell Interaction Analyses*: Genes expressed in more than three spots were subjected to cell–cell interaction analyses using stLearn^[^ ^51^ ^]^ (version: 0.4.0), as it could use both gene expression and spatial location information, which was performed separately for each sample. A permutation test was conducted 2000 times to determine the significance of cell interactions.

*Signaling Pathway Activities Examination*: To determine the activity of signaling pathways in the ST sample, the pathway activity scores for each spot were assessed using the Seurat function AddModuleScore, relying on the pathway genes documented in the KEGG database.

*Immunohistochemistry*: All dorsal skin samples were embedded in paraffin and sectioned at 4 mm to generate longitudinal sections. The immunofluorescence staining was performed as described by Jiang et al.^[^ ^7^ ^]^ Sections were imaged using a TCS SP5 confocal microscope (Leica, Wetzlar, Germany) and three randomly selected images were used for analysis. Details of the primary antibodies are displayed in Table S6 (Supporting Information).

### Statistical Analysis

All statistical analyses were performed using R software. The results are presented as the mean ± SD. The significance of differences was determined using the two‐tailed independent Student's *t‐*test for comparisons between two independent groups, and differences with *P* < 0.05 were considered statistically significant. In CytoTRACE analyses, the Kruskal‐Wallis nonparametric analysis was employed with Duncan's multiple comparison tests to determine statistical significance. Differences with *P* < 0.05 were considered statistically significant.

## Conflict of Interest

The authors declare no conflict of interest.

## Author Contributions

Y.W. and Y.J. contributed equally to this work. X.D., Y.J., and Y.W. designed the study. Y.W., Y.J., and G.N. analyzed the scRNA‐seq and ST data. Y.J. and Y.W. wrote the manuscript. Y.W., Y.J., G.N., B.B., and X.D. reviewed and edited the manuscript. The other authors assisted with the experiments and discussed the results. All the authors have read and approved the final version of the manuscript.

## Supporting information

Supporting Information

Supporting Information

Supporting Information

Supporting Information

## Data Availability

The data that support the findings of this study are available from the corresponding author upon reasonable request.
