# Supplementary material for: Integrating Single‐Cell and Spatial Transcriptomics Reveals Heterogeneity of Early Pig Skin Development and a Subpopulation with Hair Placode Formation
Source: Adv Sci (Weinh). 2024 Apr 1;11(20):2306703. doi: 10.1002/advs.202306703 (PMC11132071; doi:10.1002/advs.202306703)
Supplement: Supplementary file 1 — Supporting Information [file ADVS-11-2306703-s001.pdf]

ADVANCED SCIENCE
Open Access

Supporting Information

for *Adv. Sci.*, DOI 10.1002/advs.202306703

Integrating Single-Cell and Spatial Transcriptomics Reveals Heterogeneity of Early Pig Skin Development and a Subpopulation with Hair Placode Formation

*Yi Wang, Yao Jiang, Guiyan Ni, Shujuan Li, Brad Balderson, Quan Zou, Huatao Liu, Yifan Jiang, Jingchun Sun and Xiangdong Ding**

Supporting Information

Integrating Single-Cell and Spatial Transcriptomics Reveals Heterogeneity of Early Pig Skin Development and a Subpopulation with Hair Placode Formation

Yi Wang[†]*, Yao Jiang*[†]*, Guiyan Ni, Shujuan Li, Brad Balderson, Quan Zou, Huatao Liu, Yifan Jiang, Jingchun Sun, Xiangdong Ding*[*]

This additional file includes:

Supplemental Figures 1-10.

Supplemental Tables 1-6.

References (1 to 46) (these refer only to references in the additional file)

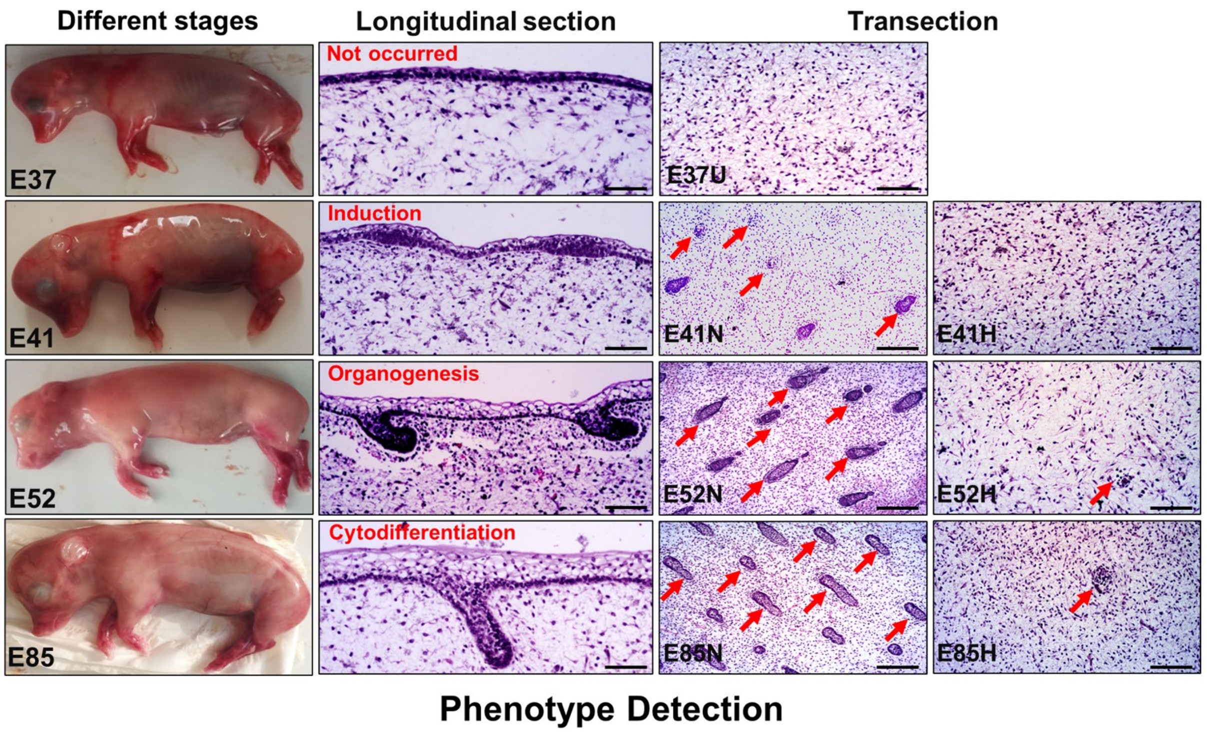

Figure S1. Phenotype detection.

Skin biopsies were collected at four stages of hair follicle morphogenesis (i.e., E37, E41, E52, and E85) from seven pig fetuses. Biopsies were used for phenotype identification, which determined three normal pigs, three hairless pigs, and one inconclusive (unknown). The hair placode, the initial structure of the hair follicle, becomes observable during the induction stage. U: Unknown; N: normal; H: hairless.

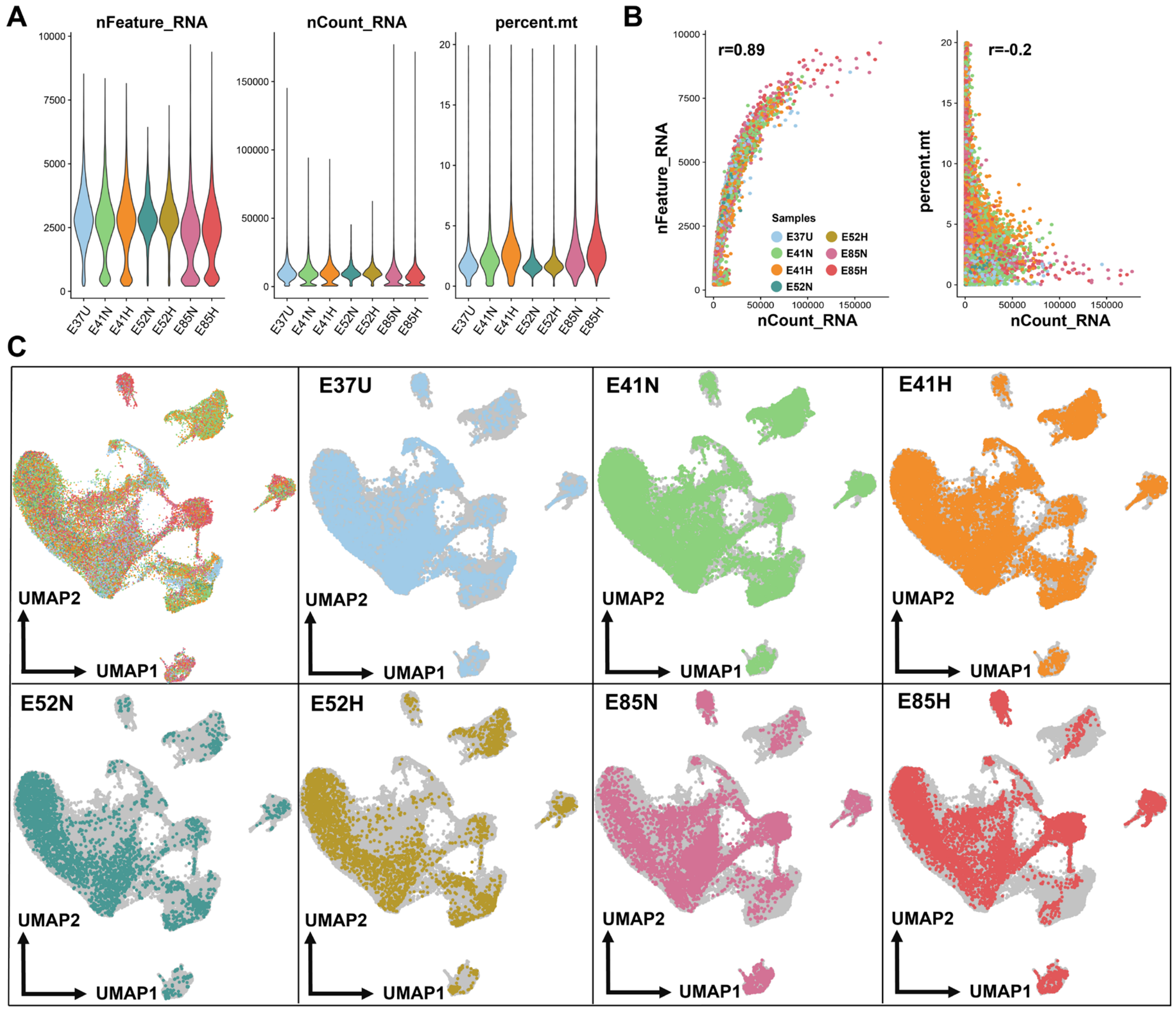

Figure S2. Quality control and integration of single-cell data.

(A) Violin plot displaying the number of RNA features, RNA counts, and percentage of mitochondrial genes of each single-cell transcriptome sequencing sample.

(B) Pair-wised relationship between RNA counts, RNA features, and percentage of mitochondrial genes per sample. Generally, the more the RNA counts, the more the features are detected. Cells with a proportion of mitochondrial genes greater than 20% were filtered out.

(C) UMAP plots labeled by samples at different developmental time points after integration. U: Unknown; N: normal; H: hairless.

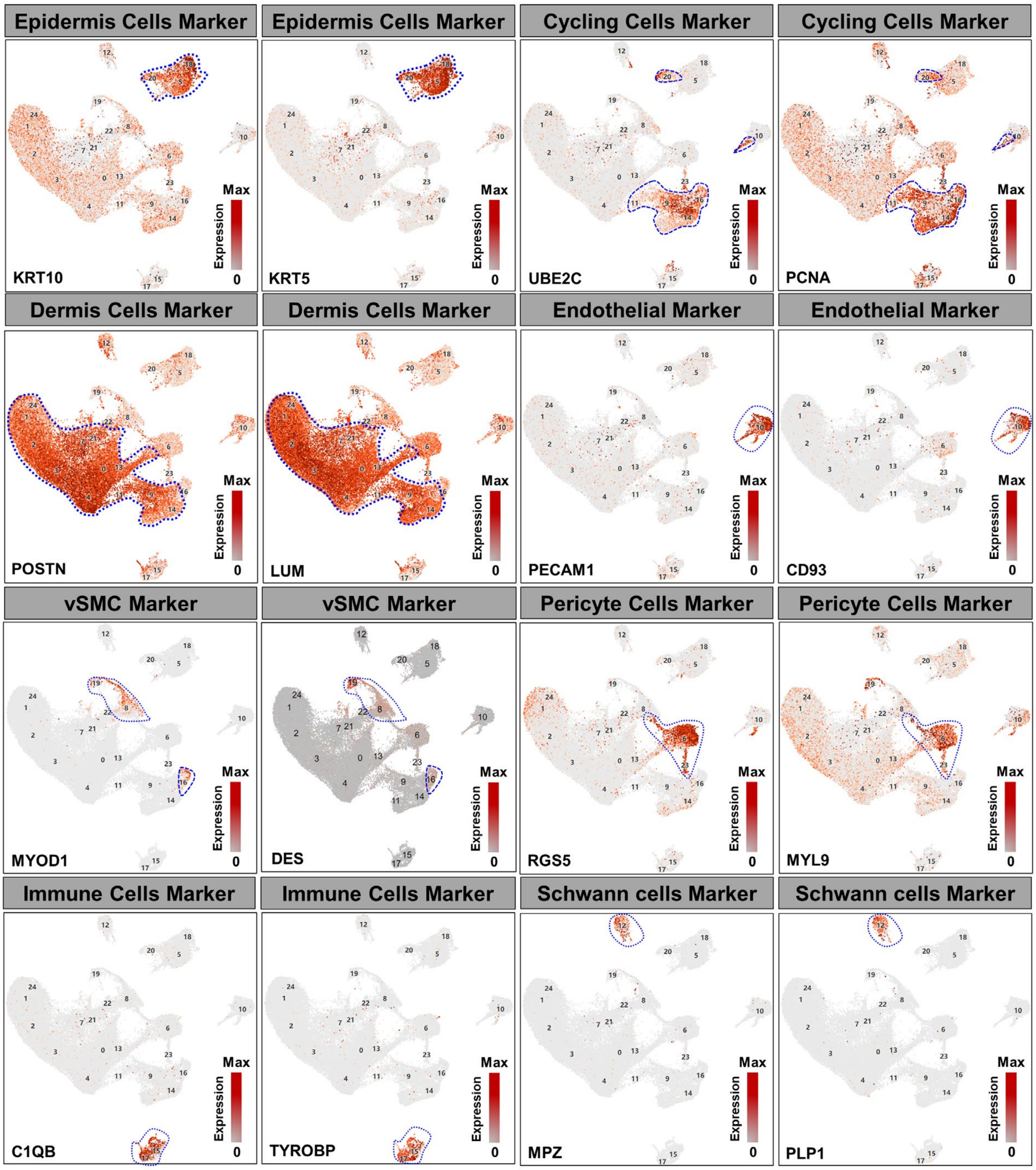

Figure S3. Visualization of canonical marker gene expression in the UMAP plot of all single cells.

Epidermis cells markers: *KRT10*, *KRT5*; Cycling cells markers: *UBE2C*, *PCNA*; Dermis cells markers: *POSTN*, *LUM*; Endothelial markers: *PECAM1*, *CD93*; vSMC (vascular smooth muscle cell) markers: *MYOD1*, *DES*; Pericyte cells markers: *RGS5*, *MYL9*; Immune cells markers: *C1QB*, *TYROBP*; Schwann cells marker: *MPZ*, *PLP1*; The blue dotted box in each UMAP plot represents the characteristic region of high expression marker genes.

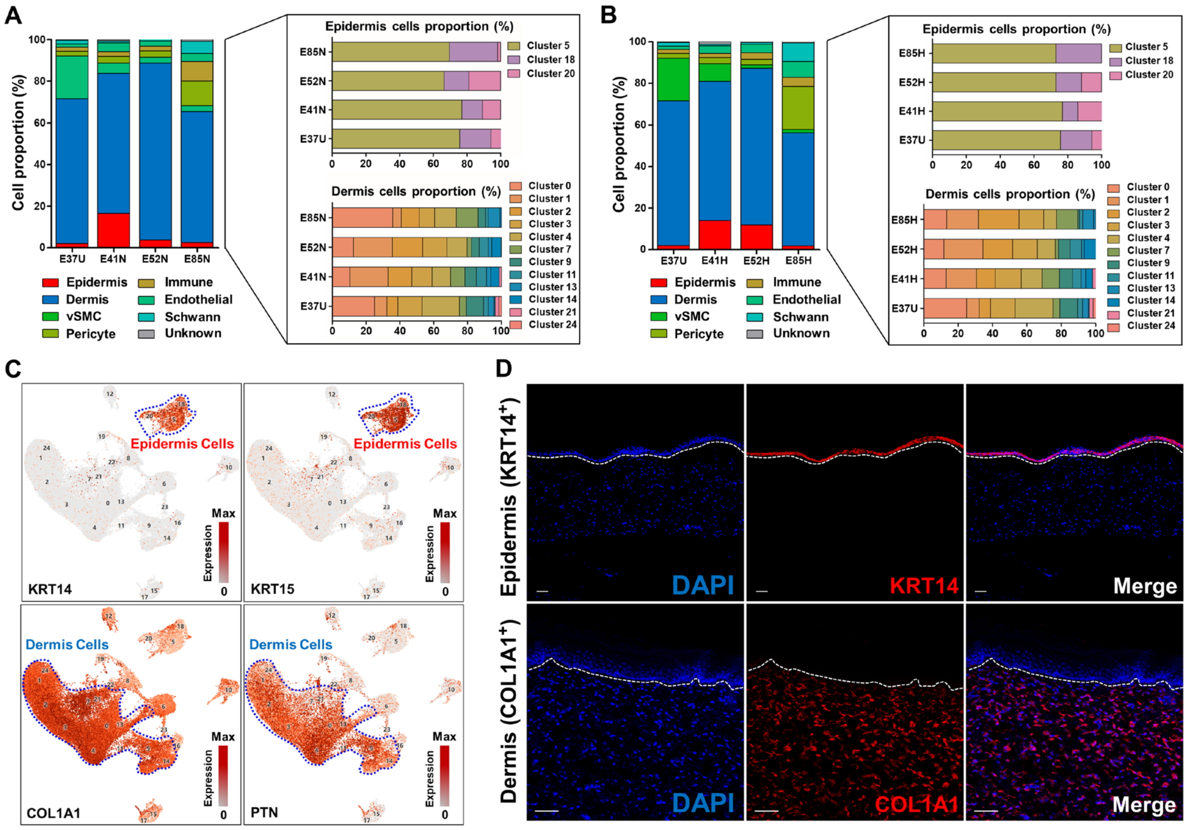

Figure S4. The proportions of cell types at different time points and validation of canonical marker genes of epidermis and dermis.

(A) The proportions of all cell lineages (left panel) and epidermis and dermis cell clusters (right panel) at different time points (E37, E41, E52, and E85) of normal samples.

(B) Bar charts showing the proportions of all cell lineages (left panel) and epidermis and dermis cell clusters (right panel) at different time points (E37, E41, E52, and E85) of hairless pig fetuses.

(C) UMAP plots showing the marker genes of epidermis (*KRT14*/*KRT15*) and dermis (*COL1A1*/*PTN*) cell subtypes. The normalized expression level for each cell is color-coded (red) and overlaid onto the UMAP plots.

(D) Immunofluorescence (IF) verifying *KRT14* in epidermis and *COL1A1* in dermis from E41 normal pig fetus skin. Scale bar = 50μm. U: Unknown; N: normal; H: hairless.

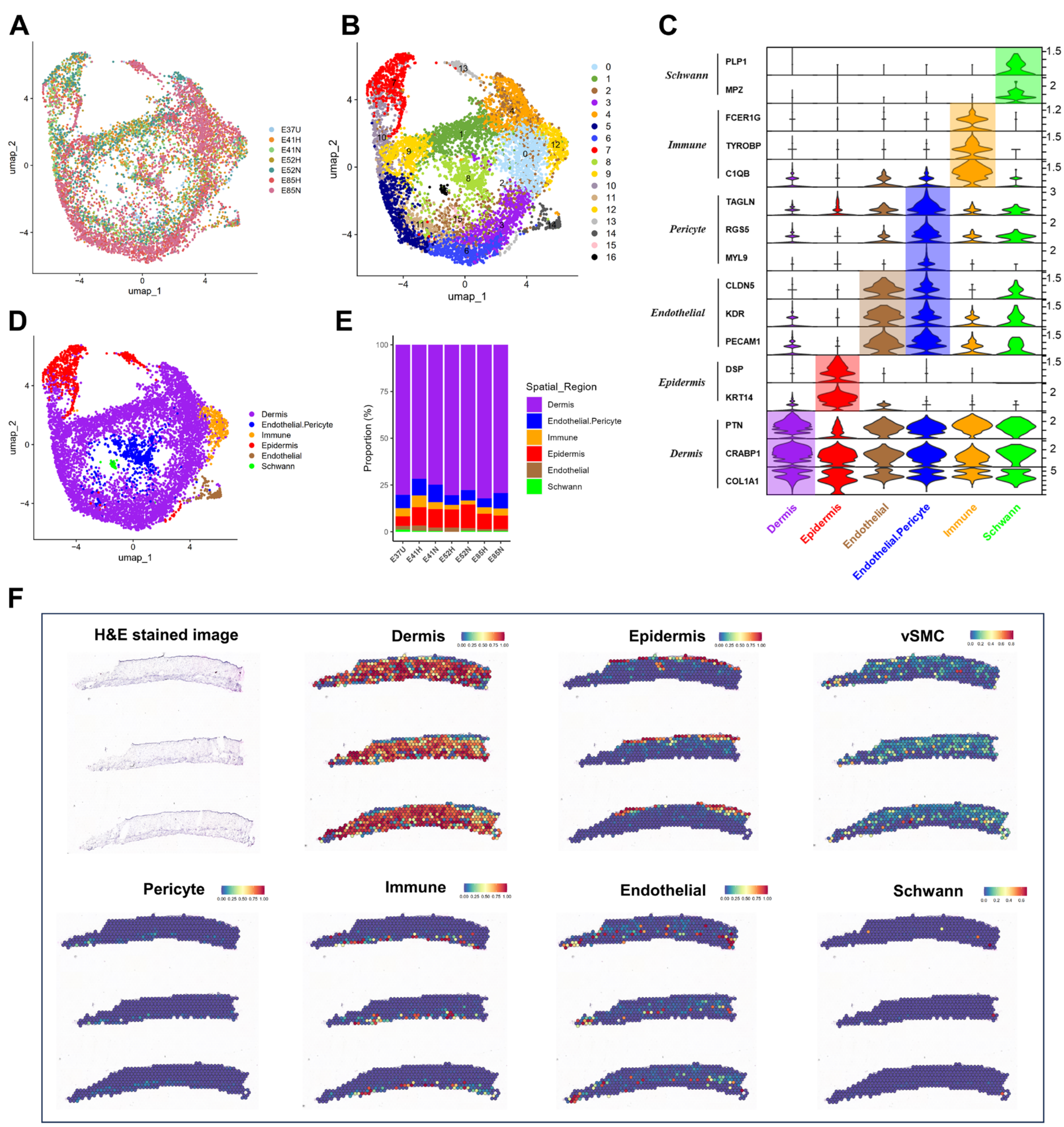

Figure S5. Characterization of cell types in pig fetuses' skin tissues in spatial transcriptomics.

(A) UMAP plot labeled by samples after integration.

(B) UMAP visualization of 8,367 spots colored by cluster.

(C) Violin plots of representative marker genes for annotating six cell types in ST. The y-axis represents characteristic marker genes for different cell types while the x-axis represents six cell types.

(D) UMAP plot of six cell types annotated by classic marker genes.

(E) Percent composition plots displaying the relative abundance of each cell type by sample.

(F) Integration of scRNA-seq dataset with ST sample. Histological image and spatial feature plots displaying spot-based enrichment of each reference cell type from scRNA-seq. Spatial plots depicting the fine map of seven cell types from scRNA-seq in ST using a representative sample (E41H).

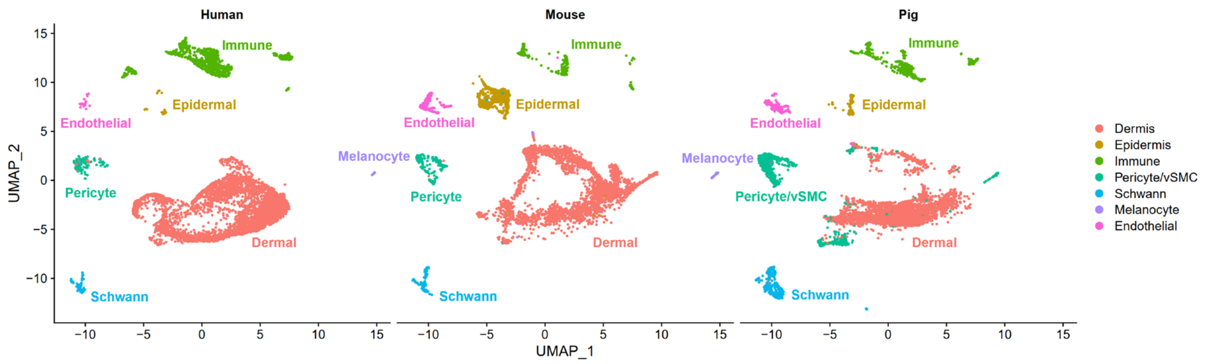

Figure S6. The UMAP plots splitting by species shows the distribution of conserved cell types.

Conserved cell types across species are depicted in a uniform color.

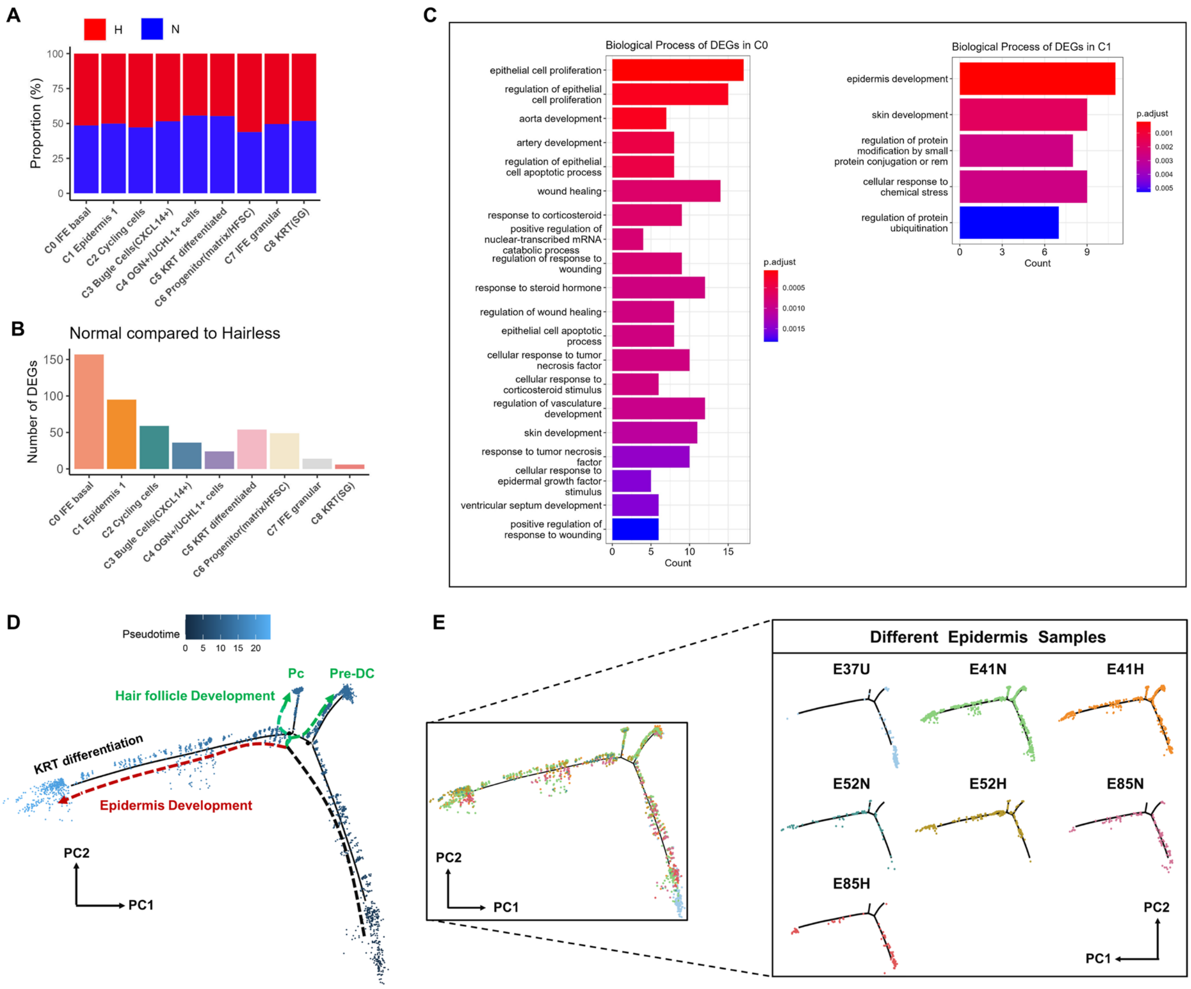

Figure S7. Differential analysis between normal and hairless groups and pseudotime ordering of the epidermal subtypes.

(A) The relative proportions of cells from the hairless (H) and normal (N) pigs within each cell subtype.

(B) The number of DEGs comparing normal and hairless samples within each cell subtype.

(C) Analysis of the biological processes associated with the DEGs comparing normal and hairless samples within the cell subtypes (left: C0 IFE basal; right: C1 Epidermis 1).

(D) Pseudotime ordering analysis of all nine epidermis cell subtypes, colored by pseudotime. The black dashed line represents common development procession; the green and red dashed lines represent hair follicle development and epidermis development, respectively.

(E) The pseudotime ordering analysis of the epidermis subtypes, colored coded by samples.

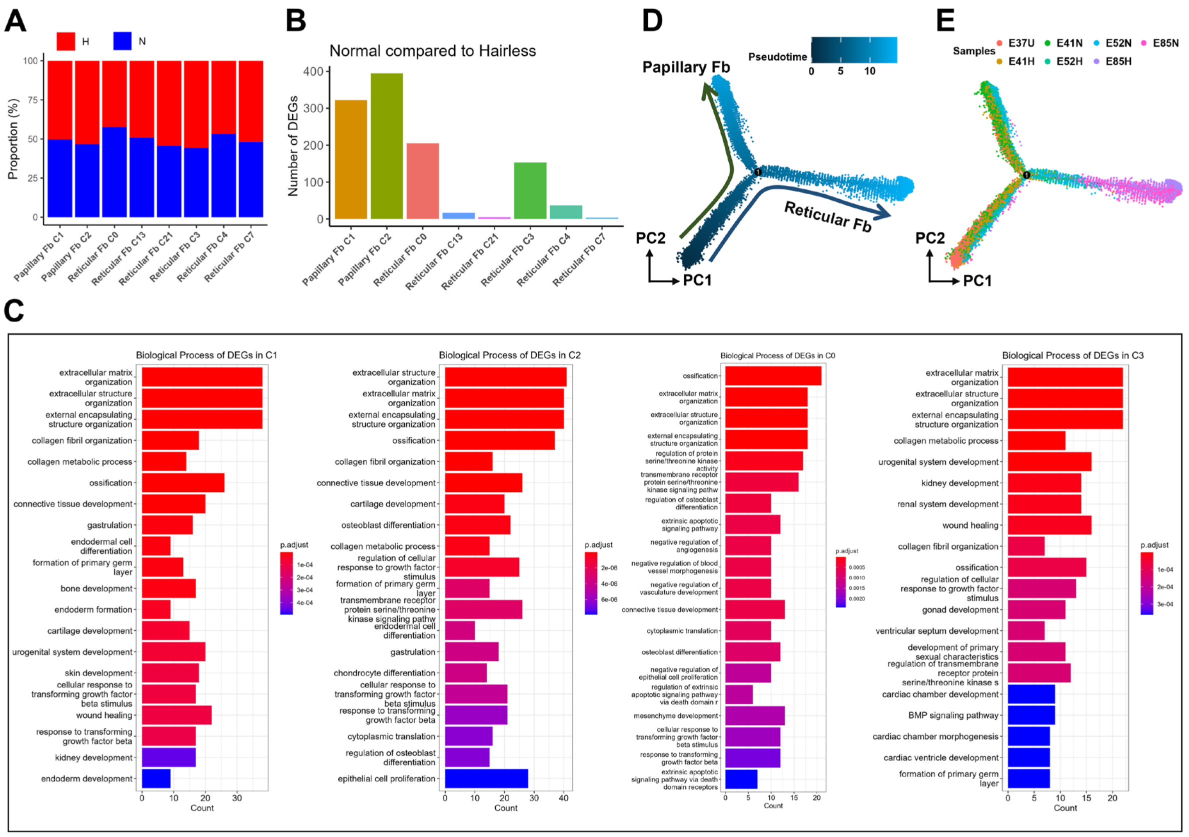

Figure S8. Differential analysis between normal and hairless groups and pseudotime ordering of the dermal subtypes.

(A) The relative proportions of samples from the hairless (H) and normal (N) pigs within each cell subtype's cluster.

(B) The number of DEGs comparing normal and hairless samples within each cell subtype's cluster.

(C) Analysis of the biological processes associated with the DEGs comparing normal and hairless samples within the cell subtypes (Papillary Fb C1; Papillary Fb C2; Reticular Fb C0; Reticular Fb C3).

(D) Pseudotime ordering analysis of the dermis subtypes, colored by pseudotime. The solid black lines with arrows reflect papillary fibroblast development and reticular fibroblast development, respectively.

(E) The pseudotime ordering trajectory of the dermis subtypes, colored by samples.

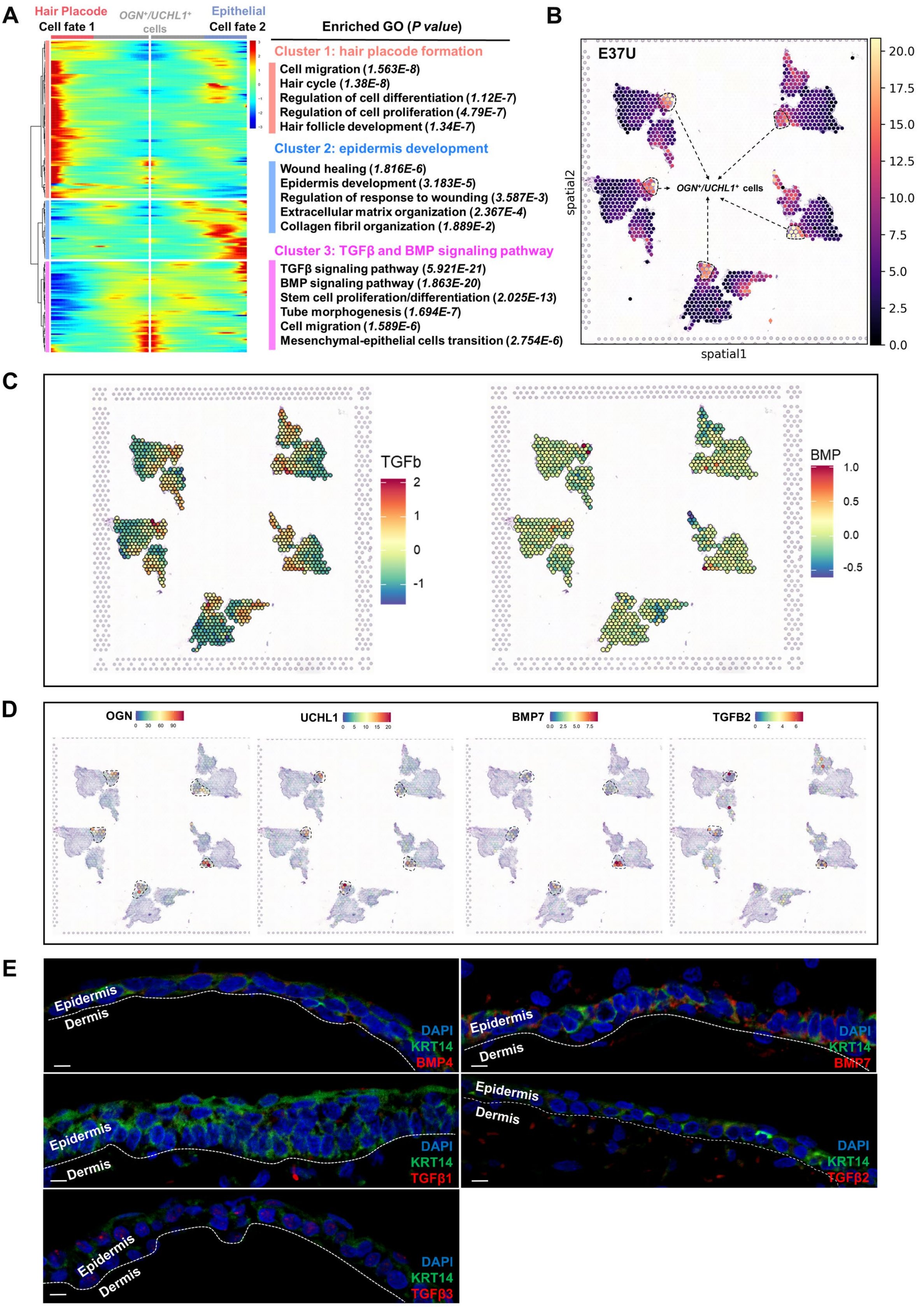

Figure S9. The differentially expressed gene dynamics and spatial validation of hair placode progenitor origins.

(A) The heatmap of DEGs (differentially expressed genes) dynamics towards cell fates of hair placode and epithelial development along the pseudotime. The DEGs are clustered into three gene sets with the GO enrichment analysis.

(B) Spatial plots showing cell abundance (color intensity) for *OGN*$^+$/*UCHL1*$^+$ cells at E37U.

(C) Pathway activity prediction on spatial transcriptomics data at E37U. TGFβ signaling pathway (left) and BMP signaling pathway (right).

(D) Spatial feature plots depicting the regions of high expression abundance for selected genes at E37U. OGN and UCHL1: the marker genes of *OGN*$^+$/*UCHL1*$^+$ cells. BMP7 and TGFβ2: the representative genes in BMP and TGFβ signaling pathways.

(E) Immunofluorescence (IF) verifying BMP (BMP4/7) and TGFβ (TGFβ1/2/3) signaling pathway in the epidermis of E37 fetal pig skins. The dotted line represents the boundary between the dermis and epidermis; Scale bar = 10μm.

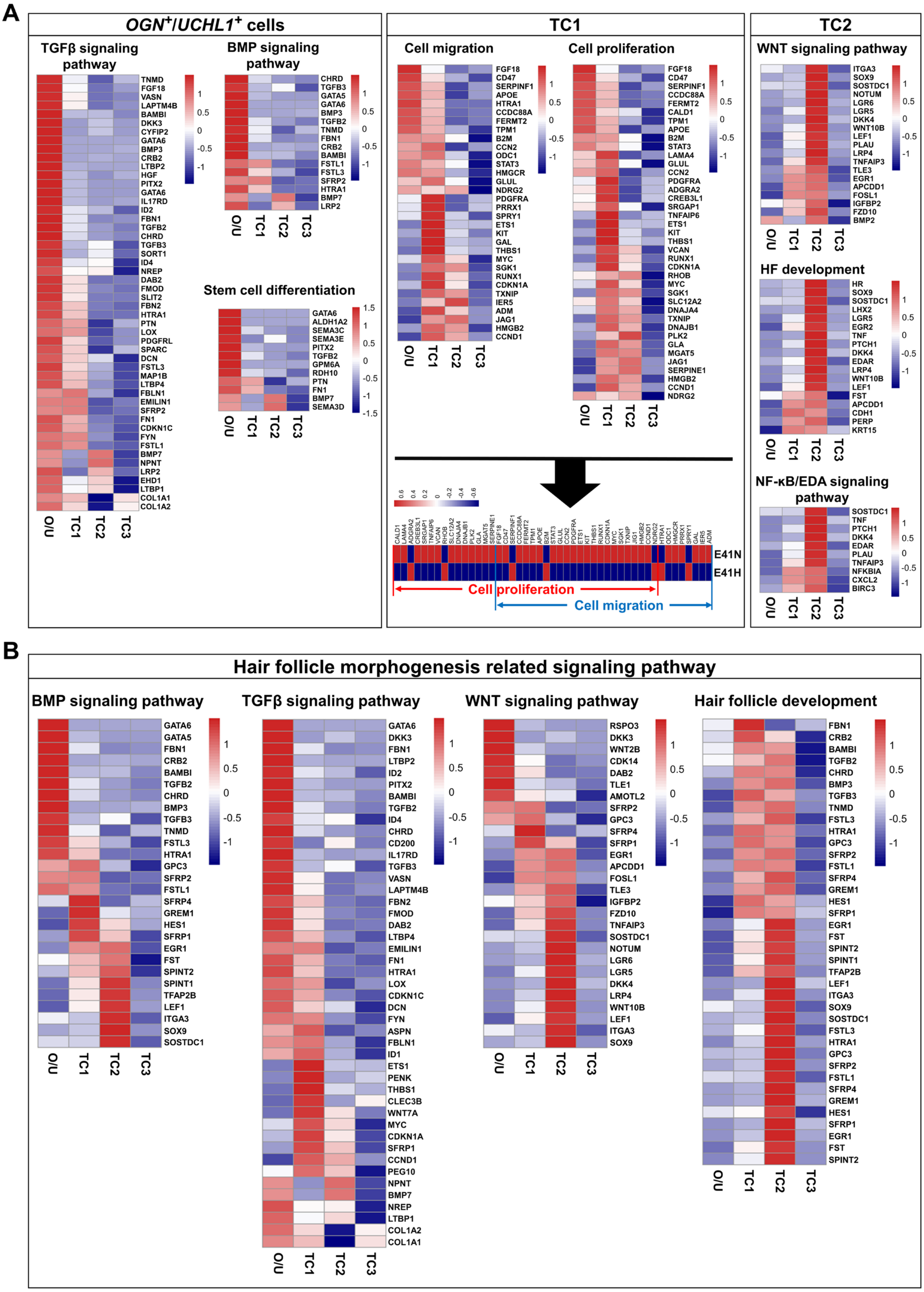

Figure S10. The transcriptional regulatory networks in the early stage of placode formation.

(A) Heatmaps of normalized expression levels for biology processions associated genes in *OGN*$^{+}$/*UCHL1*$^{+}$ cells, TC1 and TC2; The differentially expressed genes related to cell proliferation and migration between normal and hairless samples at E41 are shown at the bottom

of the TC1 panel.
(B) Heatmaps of relative expression levels for signaling pathways associated with hair follicle morphogenesis development in each cell subtype of *OGN*$^{+}$/*UCHL1*$^{+}$ cells, TC1, TC2, and TC3.

Table S1. The summary of single cell datasets quality metrics identified by Cell Ranger.

Sample info.	E37U	E41N	E41H	E52N	E52H	E85N	E85H
Estimated cells	9,224	16,740	12,718	3,224	3,228	7,710	8,515
Valid barcodes	98.2%	98.2%	98.0%	98.4%	98.4%	98.6%	98.5%
Mean reads/cells	94,148	57,714	71,920	166,363	178,469	56,963	52,146
Median genes/cell	3,512	2,891	3,031	3,787	3,887	2,230	2,345
Total genes	17,965	18,238	18,119	17,143	17,308	17,407	17,466
Reads mapped to genome	95.1%	95.3%	95.4%	95.6%	95.6%	95.3%	94.8%
Reads mapped confidently to transcriptome	45.1%	44.1%	45.0%	47.8%	46.9%	48.5%	42.0%

Table S2. The summary of spatial transcriptome datasets quality metrics identified by Space Ranger.

Sample	E37U	E41N	E41H	E52N	E52H	E85N	E85H
Number of Spots Under Tissue	794	932	1156	1148	935	1834	1625
Mean Reads/Spot	439,630	377,450	324,690	328,140	343,070	191,460	248,000
Mean Reads Under Tissue/Spot	231,200	209,040	199,670	170,010	175,060	144,950	159,770
Fraction of Spots Under Tissue	15.91%	18.67%	23.16%	23.00%	18.73%	36.74%	32.55%
Median Genes/Spot	3,476	1,486	2,300	2,284	2,347	3,293	3,843
Median UMI Counts/Spot	11,684	3,956	6,692	6,082	6,569	12,207	15,749
Valid Barcodes	97.28%	97.41%	97.52%	97.05%	97.11%	97.47%	96.71%
Valid UMIs	99.97%	99.97%	99.98%	99.98%	99.96%	99.95%	99.96%

Table S3. The canonical cell-type markers of seven major cell lineages in pig skin.

Cell lineages	Marker genes	References
Epidermis cells	*KRT10*$^+$, *KRT15*$^+$, *KRT14*$^+$, *KRT5*$^+$, *EPCAM*$^+$, *DSG3*$^+$	(Fuchs 1991; Moll et al. 2008; Bragulla & Homberger 2009; Gaiser et al. 2012; Alibardi 2016; Bharathan & Dickinson 2019)
Dermis cells	*COL1A1*$^+$, *CRABP1*$^+$, *SFRP2*$^+$, *PTN*$^+$, *PDGFRA*$^+$	(Zhang et al. 2013; Kong et al. 2016; Niu et al. 2019; Phan et al. 2021; Yao et al. 2022)
vSMC cells	*MYOD1*$^+$, *MYF6*$^+$, *DES*$^+$	(Dias et al. 1994; Talbot & Maves 2016; Maeng et al. 2021)
Immune cells	*C1QB*$^+$, *TYROBP*$^+$, *FCER1G*$^+$	(Chou et al. 2022; Feng et al. 2022; Yang et al. 2022)
Endothelial cells	*PECAM1*$^+$, *CD93*$^+$	(Dunleavey et al. 2014; Shehata et al. 2022)
Pericyte cells	*RGS5*$^+$, *MYL9*$^+$	(Ge et al. 2020; Dasgupta et al. 2021)
Schwann cells	*MPZ*$^+$, *PLP1*$^+$	(Chang et al. 2019; Xie et al. 2019)

Table S4. The canonical cell-type markers of nine subclasses in pig epidermis cells.

Epidermis cell subtypes	Marker genes	References
IFE basal	*COL17A1*$^+$, *KRT14*$^+$	(Natsuga et al. 2019; Koenig et al. 2020)
Epidermis 1	*KRT15*$^+$, *PDGFA*$^+$	(Rishikaysh et al. 2014; Wiener et al. 2020)
Cycling cells	*UBE2C*$^+$, *PCNA*$^+$	(Jurikova et al. 2016; Yuan et al. 2022)
Bugle Cells	*CXCL14*$^+$, *HBB*$^+$	(Yoo et al. 2010; Ge *et al.* 2020)
KRT differentiated	*KRTDAP*$^+$, *SBSN*$^+$	(Oomizu et al. 2000; Nouioui et al. 2018; Ge et al. 2021)
Progenitor (matrix/HFSC)	*SOX9*$^+$, *LEF1*$^+$, *EDAR*$^+$	(Nowak et al. 2008; Rishikaysh *et al.* 2014; Kloepper et al. 2015; Saxena et al. 2019; Jiang et al. 2021; Jiang et al. 2022)
IFE granular	*CALML5*$^+$, *NOTCH1*$^+$	(Ge *et al.* 2020; Ge *et al.* 2021; Yoshioka et al. 2021)
KRT(SG)	*MGST1*$^+$, *SPINK5*$^+$	(Komatsu et al. 2003; Meyer-Hoffert et al. 2010; Joost et al. 2016)

Table S5. The canonical cell-type markers used for annotation of pig dermis cell subtypes.

Dermis cell subtypes	Marker genes	References
Papillary fibroblasts	*APCDD1*$^{+}$, *AXIN2*$^{+}$,	(Philippeos et al. 2018; Solé-Boldo et al. 2020)
Reticular fibroblasts	*MFAP5*$^{+}$, *COL11A1*$^{+}$,	(Philippeos *et al.* 2018; Solé-Boldo *et al.* 2020)
Common blast progenitor	*PDGFRA*$^{+}$, *DL1K*$^{+}$,	(Driskell et al. 2013; Lynch & Watt 2018)
Papillary dermal fibroblast progenitor	*PDGFRA*$^{+}$, *DL1K*$^{-}$, *APCDD1*$^{+}$	(Driskell *et al.* 2013; Lynch & Watt 2018)
Reticular dermal fibroblast progenitor	*PDGFRA*$^{+}$, *DL1K*$^{+}$, *COL11A1*$^{+}$	(Driskell *et al.* 2013; Lynch & Watt 2018)

Table S6. The information on antibodies in this study.

Antibody	Period	Expression region	Brand	Source species	Reaction species	Catalog number	Concentration
KRT14	All	Epidermis	Santa	Mouse	Human, Mouse, Rat	sc-47697	1:500
COL1A1	All	Dermis	Abmart	Rabbit	Human, Mouse, Rat, Cow	P28373-B2	1:500
LEF1	E41	Pc	Abcam	Rabbit	Human, Mouse, Rat	ab137872	1:500
SOX9	E52	Hair peg	Abcam	Rabbit	Human, Mouse, Rat	ab185966	1:800
BMP4	E37	O/U cells (Epidermis)	Abmart	Rabbit	Human	T55190S	1:300
BMP7	E37	O/U cells (Epidermis)	Abmart	Rabbit	Human, Mouse, Rat, Chicken, Dog, Monkey, Pig, Rabbit	PA1101S	1:100
BMP7	E37	O/U cells (Epidermis)	Santa	Mouse	Human	sc-517294	1:100
TGFβ1	E37	O/U cells (Epidermis)	Abmart	Rabbit	Human, Mouse, Rat, Bovine, Dog, Pig, Sheep	PA2154S	1:100
TGFβ2	E37	O/U cells (Epidermis)	Abcam	Rabbit	Human, Mouse	ab208687	1:500
TGFβ2	E37	O/U cells (Epidermis)	Santa	Mouse	Human, Mouse, Rat	sc-374658	1;100
TGFβ3	E37	O/U cells (Epidermis)	Abcam	Rabbit	Human, Mouse, Rat, Rabbit	ab15537	1:500
OGN	E37	O/U cells (Epidermis)	Proteintech	Rabbit	Human, Mouse, Rat	12755-1-AP	1:100
UCHL1	E37	O/U cells (Epidermis)	Boster	Rabbit	Human, Mouse, Rat	BM4990	1:100

Reference

1. Alibardi L. (2016) Sauropsids Cornification is Based on Corneous Beta-Proteins, a Special Type of Keratin-Associated Corneous Proteins of the Epidermis. J Exp Zool B Mol Dev Evol 326, 338-51.
2. Bharathan N.K. & Dickinson A.J.G. (2019) Desmoplakin is required for epidermal integrity and morphogenesis in the Xenopus laevis embryo. Dev Biol 450, 115-31.
3. Bragulla H.H. & Homberger D.G. (2009) Structure and functions of keratin proteins in simple, stratified, keratinized and cornified epithelia. J Anat 214, 516-59.
4. Chang E.H., Mo W.M., Doo H.M., Lee J.S., Park H.T., Choi B.O. & Hong Y.B. (2019) Aminosalicylic acid reduces ER stress and Schwann cell death induced by MPZ mutations. Int J Mol Med 44, 125-34.
5. Chou C., Zhang X., Krishna C., Nixon B.G., Dadi S., Capistrano K.J., Kansler E.R., Steele M., Han J., Shyu A., Zhang J., Stamatiades E.G., Liu M., Li S., Do M.H., Edwards C., Kang D.S., Chen C.T., Wei I.H., Pappou E.P., Weiser M.R., Garcia-Aguilar J., Smith J.J., Leslie C.S. & Li M.O. (2022) Programme of self-reactive innate-like T cell-mediated cancer immunity. Nature 605, 139-45.
6. Dasgupta S., Ghosh T., Dhar J., Bhuniya A., Nandi P., Das A., Saha A., Das J., Guha I., Banerjee S., Chakravarti M., Dasgupta P.S., Alam N., Chakrabarti J., Majumdar S., Chakrabarti P., Storkus W.J., Baral R. & Bose A. (2021) RGS5-TGFbeta-Smad2/3 axis switches pro- to anti-apoptotic signaling in tumor-residing pericytes, assisting tumor growth. Cell Death Differ 28, 3052-76.
7. Dias P., Dilling M. & Houghton P. (1994) The molecular basis of skeletal muscle differentiation. Semin Diagn Pathol 11, 3-14.
8. Driskell R.R., Lichtenberger B.M., Hoste E., Kretzschmar K., Simons B.D., Charalambous M., Ferron S.R., Herault Y., Pavlovic G., Ferguson-Smith A.C. & Watt F.M. (2013) Distinct fibroblast lineages determine dermal architecture in skin development and repair. Nature 504, 277-81.
9. Dunleavey J.M., Xiao L., Thompson J., Kim M.M., Shields J.M., Shelton S.E., Irvin D.M., Brings V.E., Ollila D.W., Brekken R.A., Dayton P.A., Melero-Martin J.M. & Dudley A.C. (2014) Vascular channels formed by subpopulations of PECAM1+ melanoma cells. Nat Commun 5, 5200.
10. Feng C., Shan M., Xia Y., Zheng Z., He K., Wei Y., Song K., Meng T., Liu H., Hao Y., Liang Z., Wang Y. & Huang Y. (2022) Single-cell RNA sequencing reveals distinct immunology profiles in human keloid. Front Immunol 13, 940645.
11. Fuchs E. (1991) Keratin genes, epidermal differentiation and animal models for the study of human skin diseases. Biochem Soc Trans 19, 1112-5.
12. Gaiser M.R., Lammermann T., Feng X., Igyarto B.Z., Kaplan D.H., Tessarollo L., Germain R.N. & Udey M.C. (2012) Cancer-associated epithelial cell adhesion molecule (EpCAM; CD326) enables epidermal Langerhans cell motility and migration in vivo. Proc Natl Acad Sci U S A 109, E889-97.
13. Ge W., Tan S.J., Wang S.H., Li L., Sun X.F., Shen W. & Wang X. (2020) Single-cell Transcriptome Profiling reveals Dermal and Epithelial cell fate decisions during Embryonic Hair Follicle Development. Theranostics 10, 7581-98.
14. Ge W., Zhang W., Zhang Y., Zheng Y., Li F., Wang S., Liu J., Tan S., Yan Z., Wang L., Shen W., Qu L. & Wang X. (2021) A Single-cell Transcriptome Atlas of Cashmere Goat Hair Follicle Morphogenesis. Genomics Proteomics Bioinformatics 19, 437-51.

15. Jiang Y., Liu H., Zou Q., Li S. & Ding X. (2022) miR-29a-5p Inhibits Prenatal Hair Placode Formation Through Targeting EDAR by ceRNA Regulatory Network. Front Cell Dev Biol 10, 902026.
16. Jiang Y., Zou Q., Liu B., Li S., Wang Y., Liu T. & Ding X. (2021) Atlas of Prenatal Hair Follicle Morphogenesis Using the Pig as a Model System. Front Cell Dev Biol 9, 721979.
17. Joost S., Zeisel A., Jacob T., Sun X., La Manno G., Lonnerberg P., Linnarsson S. & Kasper M. (2016) Single-Cell Transcriptomics Reveals that Differentiation and Spatial Signatures Shape Epidermal and Hair Follicle Heterogeneity. Cell Syst 3, 221-37 e9.
18. Jurikova M., Danihel L., Polak S. & Varga I. (2016) Ki67, PCNA, and MCM proteins: Markers of proliferation in the diagnosis of breast cancer. Acta Histochem 118, 544-52.
19. Kloepper J.E., Baris O.R., Reuter K., Kobayashi K., Weiland D., Vidali S., Tobin D.J., Niemann C., Wiesner R.J. & Paus R. (2015) Mitochondrial function in murine skin epithelium is crucial for hair follicle morphogenesis and epithelial-mesenchymal interactions. J Invest Dermatol 135, 679-89.
20. Koenig U., Robenek H., Barresi C., Brandstetter M., Resch G.P., Groger M., Pap T. & Hartmann C. (2020) Cell death induced autophagy contributes to terminal differentiation of skin and skin appendages. Autophagy 16, 932-45.
21. Komatsu N., Takata M., Otsuki N., Toyama T., Ohka R., Takehara K. & Saijoh K. (2003) Expression and localization of tissue kallikrein mRNAs in human epidermis and appendages. J Invest Dermatol 121, 542-9.
22. Kong R., Cui Y., Fisher G.J., Wang X., Chen Y., Schneider L.M. & Majmudar G. (2016) A comparative study of the effects of retinol and retinoic acid on histological, molecular, and clinical properties of human skin. J Cosmet Dermatol 15, 49-57.
23. Lynch M.D. & Watt F.M. (2018) Fibroblast heterogeneity: implications for human disease. J Clin Invest 128, 26-35.
24. Maeng G., Das S., Greising S.M., Gong W., Singh B.N., Kren S., Mickelson D., Skie E., Gafni O., Sorensen J.R., Weaver C.V., Garry D.J. & Garry M.G. (2021) Humanized skeletal muscle in MYF5/MYOD/MYF6-null pig embryos. Nat Biomed Eng 5, 805-14.
25. Meyer-Hoffert U., Wu Z., Kantyka T., Fischer J., Latendorf T., Hansmann B., Bartels J., He Y., Glaser R. & Schroder J.M. (2010) Isolation of SPINK6 in human skin: selective inhibitor of kallikrein-related peptidases. J Biol Chem 285, 32174-81.
26. Moll R., Divo M. & Langbein L. (2008) The human keratins: biology and pathology. Histochem Cell Biol 129, 705-33.
27. Natsuga K., Watanabe M., Nishie W. & Shimizu H. (2019) Life before and beyond blistering: The role of collagen XVII in epidermal physiology. Exp Dermatol 28, 1135-41.
28. Niu X., Li J., Zhao X., Wang Q., Wang G., Hou R., Li X., An P., Yin G. & Zhang K. (2019) Dermal mesenchymal stem cells: a resource of migration-associated function in psoriasis? Stem Cell Res Ther 10, 54.
29. Nouioui M.A., Araoud M., Milliand M.L., Bessueille-Barbier F., Amira D., Ayouni-Derouiche L. & Hedhili A. (2018) Evaluation of the status and the relationship between essential and toxic elements in the hair of occupationally exposed workers. Environ Monit Assess 190, 731.
30. Nowak J.A., Polak L., Pasolli H.A. & Fuchs E. (2008) Hair follicle stem cells are specified and function in early skin morphogenesis. Cell Stem Cell 3, 33-43.
31. Oomizu S., Sahuc F., Asahina K., Inamatsu M., Matsuzaki T., Sasaki M., Obara M. & Yoshizato K. (2000) Kdap, a novel gene associated with the stratification of the epithelium. Gene 256, 19-27.

32. Phan Q.M., Sinha S., Biernaskie J. & Driskell R.R. (2021) Single-cell transcriptomic analysis of small and large wounds reveals the distinct spatial organization of regenerative fibroblasts. Exp Dermatol 30, 92-101.
33. Philippeos C., Telerman S.B., Oulès B., Pisco A.O., Shaw T.J., Elgueta R., Lombardi G., Driskell R.R., Soldin M., Lynch M.D. & Watt F.M. (2018) Spatial and Single-Cell Transcriptional Profiling Identifies Functionally Distinct Human Dermal Fibroblast Subpopulations. J Invest Dermatol 138, 811-25.
34. Rishikaysh P., Dev K., Diaz D., Qureshi W.M., Filip S. & Mokry J. (2014) Signaling involved in hair follicle morphogenesis and development. Int J Mol Sci 15, 1647-70.
35. Saxena N., Mok K.W. & Rendl M. (2019) An updated classification of hair follicle morphogenesis. Exp Dermatol 28, 332-44.
36. Shehata W.A., Maraee A.H., Tayel N., Mohamed A.S., Abd El Gayed E.M., Elsayed N., Mostafa M.I. & Bazid H.A.S. (2022) CD93 has a crucial role in pathogenesis of psoriasis. J Cosmet Dermatol 21, 1616-24.
37. Solé-Boldo L., Raddatz G., Schütz S., Mallm J.P., Rippe K., Lonsdorf A.S., Rodríguez-Paredes M. & Lyko F. (2020) Single-cell transcriptomes of the human skin reveal age-related loss of fibroblast priming. Commun Biol 3, 188.
38. Talbot J. & Maves L. (2016) Skeletal muscle fiber type: using insights from muscle developmental biology to dissect targets for susceptibility and resistance to muscle disease. Wiley Interdiscip Rev Dev Biol 5, 518-34.
39. Wiener D.J., Groch K.R., Brunner M.A.T., Leeb T., Jagannathan V. & Welle M.M. (2020) Transcriptome Profiling and Differential Gene Expression in Canine Microdissected Anagen and Telogen Hair Follicles and Interfollicular Epidermis. Genes (Basel) 11.
40. Xie M., Kamenev D., Kaucka M., Kastriti M.E., Zhou B., Artemov A.V., Storer M., Fried K., Adameyko I., Dyachuk V. & Chagin A.S. (2019) Schwann cell precursors contribute to skeletal formation during embryonic development in mice and zebrafish. Proc Natl Acad Sci U S A 116, 15068-73.
41. Yang H., Che D., Gu Y. & Cao D. (2022) Prognostic and immune-related value of complement C1Q (C1QA, C1QB, and C1QC) in skin cutaneous melanoma. Front Genet 13, 940306.
42. Yao L., Rathnakar B.H., Kwon H.R., Sakashita H., Kim J.H., Rackley A., Tomasek J.J., Berry W.L. & Olson L.E. (2022) Temporal control of PDGFRalpha regulates the fibroblast-to-myofibroblast transition in wound healing. Cell Rep 40, 111192.
43. Yoo B.Y., Shin Y.H., Yoon H.H., Seo Y.K., Song K.Y. & Park J.K. (2010) Application of mesenchymal stem cells derived from bone marrow and umbilical cord in human hair multiplication. J Dermatol Sci 60, 74-83.
44. Yoshioka H., Yamada T., Hasegawa S., Miyachi K., Ishii Y., Hasebe Y., Inoue Y., Tanaka H., Iwata Y., Arima M., Sugiura K. & Akamatsu H. (2021) Senescent cell removal via JAG1-NOTCH1 signalling in the epidermis. Exp Dermatol 30, 1268-78.
45. Yuan L., Yang Z., Zhao J., Sun T., Hu C., Shen Z. & Yu G. (2022) Pan-Cancer Bioinformatics Analysis of Gene UBE2C. Front Genet 13, 893358.
46. Zhang Q., Tao K., Huang W., Tian Y. & Liu X. (2013) Elevated expression of pleiotrophin in human hypertrophic scars. J Mol Histol 44, 91-6.
